# AP endonucleases process 5-methylcytosine excision intermediates during active DNA demethylation in *Arabidopsis*

**DOI:** 10.1093/nar/gku834

**Published:** 2014-09-16

**Authors:** Jiyoon Lee, Hosung Jang, Hosub Shin, Woo Lee Choi, Young Geun Mok, Jin Hoe Huh

**Affiliations:** Department of Plant Science, Research Institute for Agriculture and Life Sciences, and Plant Genomics and Breeding Institute, Seoul National University, Seoul 151–921, Korea

## Abstract

DNA methylation is a primary epigenetic modification regulating gene expression and chromatin structure in many eukaryotes. Plants have a unique DNA demethylation system in that 5-methylcytosine (5mC) is directly removed by DNA demethylases, such as DME/ROS1 family proteins, but little is known about the downstream events. During 5mC excision, DME produces 3′-phosphor-α, β-unsaturated aldehyde and 3′-phosphate by successive β- and δ-eliminations, respectively. The kinetic studies revealed that these 3′-blocking lesions persist for a significant amount of time and at least two different enzyme activities are required to immediately process them. We demonstrate that *Arabidopsis* AP endonucleases APE1L, APE2 and ARP have distinct functions to process such harmful lesions to allow nucleotide extension. *DME* expression is toxic to *E. coli* due to excessive 5mC excision, but expression of *APE1L* or *ARP* significantly reduces DME-induced cytotoxicity. Finally, we propose a model of base excision repair and DNA demethylation pathway unique to plants.

## INTRODUCTION

DNA methylation is a primary epigenetic modification that regulates gene expression and chromatin structure (1–3). In eukaryotes, DNA methylation often refers to the conversion of cytosine to 5-methylcytosine (5mC), which is catalyzed by DNA methyltransferases (2). Tight control of DNA methylation is crucial in plants in that it is important for many developmental processes, including gene imprinting and transposon silencing (2,4). Like most epigenetic modifications, DNA methylation can be reversible. DNA demethylation, the reverse process of DNA methylation, can be classified into two different mechanisms. Passive DNA demethylation involves inactivation or down-regulation of maintenance DNA methyltransferases, such as DNMT1 and MET1, in mammals and plants, respectively, by which the level of 5mC gradually decreases in a replication-dependent manner. By contrast, active DNA demethylation enzymatically occurs by DNA demethylases in a replication-independent manner (5).

Many lines of evidence suggest that DNA repair machineries are employed to allow active DNA demethylation in both plants and mammals. In particular, the base excision repair (BER) pathway plays an essential role in removing 5mC from DNA. According to the current models of active DNA demethylation, 5mC is directly recognized and excised in plants by specific DNA glycosylases, and its replacement with unmethylated cytosine via the BER pathway completes demethylation (6–10). However, DNA demethylation in mammals is unlikely to involve direct removal of 5mC. Instead, it begins with chemical modifications of 5mC to other bases, such as thymine by oxidative deamination or 5-hydroxymethylcytosine (5hmC), 5-formylcytosine (5fC) and 5-carboxylcytosine (5caC) by oxidation processes, which are then excised by mismatch DNA glycosylase, such as thymine DNA glycosylase (TDG) (11–16).

DEMETER (DME) is a founding member of the plant-specific DNA demethylase family which was first identified in *Arabidopsis* (17). DME and its homologs, such as ROS1, DML2 and DML3, all have 5mC DNA glycosylase activity *in vitro* (6,7,9,18,19). As bifunctional DNA glycosylases with additional apurinic/apyrimidinic (AP)-lyase activity, the DME family proteins catalyze both 5mC excision and the cleavage of a sugar-phosphate backbone via β- and δ-elimination reactions, producing 3′-phosphor-α, β-unsaturated aldehyde (3′-PUA) and 3′-phosphate, respectively. These must be processed to provide 3′-OH for subsequent polymerization. Thus, further demethylation steps may require BER machineries. In particular, AP endonucleases that act immediately downstream of DNA glycosylase are expectedly indispensable for processing such harmful lesions.

Recently, it was reported that zinc finger DNA 3′phosphoesterase (ZDP) is necessary for ROS1-mediated DNA demethylation in *Arabidopsis* (20). ZDP was found to preferentially remove the δ-elimination product 3′-phosphate at the 5mC excision site, providing 3′-OH to allow subsequent polymerization and ligation to complete 5mC replacement with unmethylated cytosine (20). However, the findings that formation of 3′-phosphate by δ-elimination is significantly a slow process raise fundamental questions regarding the biological relevance of the proposed mechanism (20,21), because the DNA strand on which 5mC excision occurs should remain open until the BER is completed, and this is extremely harmful as it inevitably prevents DNA replication and transcription (22).

In this study, we show that both DME and ROS1 5mC DNA glycosylases generate 3′-PUA as a primary 5mC excision intermediate, which needs immediate attention of DNA repair machineries. To investigate the functional roles in 5mC excision, three AP endonucleases APE1L, APE2 and ARP present in the *Arabidopsis* genome are subjected to a thorough biochemical analysis. We report both APE1L and ARP are capable of processing the 3′-blocking lesions generated by DME. In addition, we demonstrate in a heterologous bacterial system that AP endonucleases effectively process such harmful lesions that are inevitably produced during the course of 5mC excision. Our data suggest that active DNA demethylation processes in plants may require two distinct enzymatic activities and that they are coordinated to completely remove unwanted 5mC excision intermediates.

## MATERIALS AND METHODS

### DNA glycosylase assay

Oligonucleotides used in this study were synthesized from Integrated DNA Technologies (IA, USA) (Supplementary Table S1). Forty pmols of R35M oligonucleotide was radiolabeled with [γ-^32^P]ATP (6000 Ci/mmol, Perkin Elmer Life Sciences) using T4 polynucleotide kinase (Takara) and then annealed to a complementary oligonucleotide to produce double-stranded DNA substrate. *In vitro* base excision assay was performed as previously described (23). Briefly, 25 nM each of radiolabeled oligonucleotide substrates was incubated with 100 nM MBP-DMEΔ or 85 nM MBP-ROS1Δ in the glycosylase reaction buffer (10 mM Tris-HCl, pH 7.4, 50 mM NaCl, 0.5 mM DTT, 200 μg/ml bovine serum albumin (BSA)) at 37°C for 1 h. For the experiments in Figure 2, two separate reactions were performed at 37°C for 25 min and paused on ice or heat-inactivated at 65°C for 15 min, respectively, and then resumed at 37°C for additional 15.6 h. Reactions were terminated at each time point indicated in Figure 2, by adding an equal volume of stop solution (98% formamide, 10 mM ethylenediaminetetraacetic acid (EDTA), 0.2% xylen cyanol FF, 0.2% bromophenol blue) and heat-denaturing at 95°C for 10 min. Reaction products were separated on a 15% denaturing polyacrylamide gel containing 7.5 M urea and 1×TBE.

**Figure 1.**
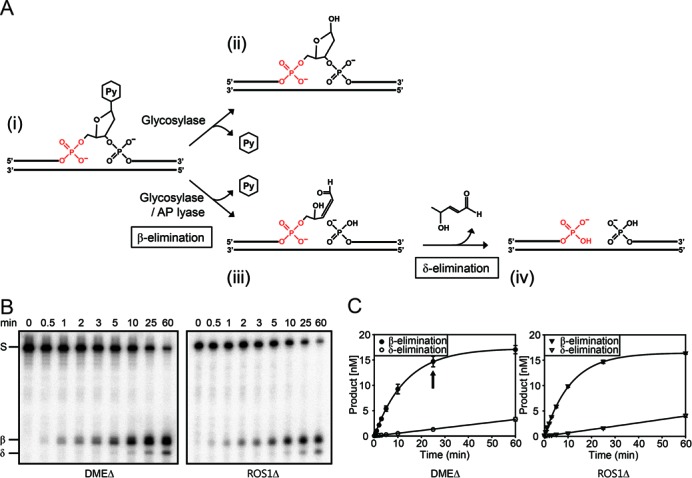
Excision of 5mC by DME and ROS1 DNA glycosylases. (A) The scheme of base excision by monofunctional or bifunctional DNA glycosylase. Upon encountering a base to remove (i), monofunctional DNA glycosylases catalyze the hydrolysis of an N-glycosidic bond between the base and a ribose sugar creating an AP site (ii). Bifunctional DNA glycosylases possess additional AP lyase activity that catalyzes the scission of the sugar phosphate backbone leaving the 3′-PUA (iii) by the process called β-elimination. Further δ-elimination process generates a 3′-phosphate (iv), which is a blocking lesion for subsequent polymerization. (B) Excision of 5mC by DME and ROS1. Purified MBP-DMEΔ or -ROS1Δ protein (23) was incubated with a radiolabeled 35-mer oligonucleotide duplex containing 5mC. Both 3′-PUA and 3′-phosphate were generated by β- and δ-elimination processes, respectively. The major intermediate formed in early reaction is a 3′-PUA (β-elimination product), and as reaction proceeds, 3′-phosphate (δ-elimination product) begins to accumulate later. (C) Relative amounts of β- and δ-elimination product accumulation. The amounts of every β- and δ-elimination product from the experiments in (B) were measured using the phosphorimager and plotted over time. An arrow indicates the time point when different temperatures (4°C or 65°C) were treated for the experiment in Figure 2. Error bars represent standard deviations from three independent experiments.

**Figure 2.**
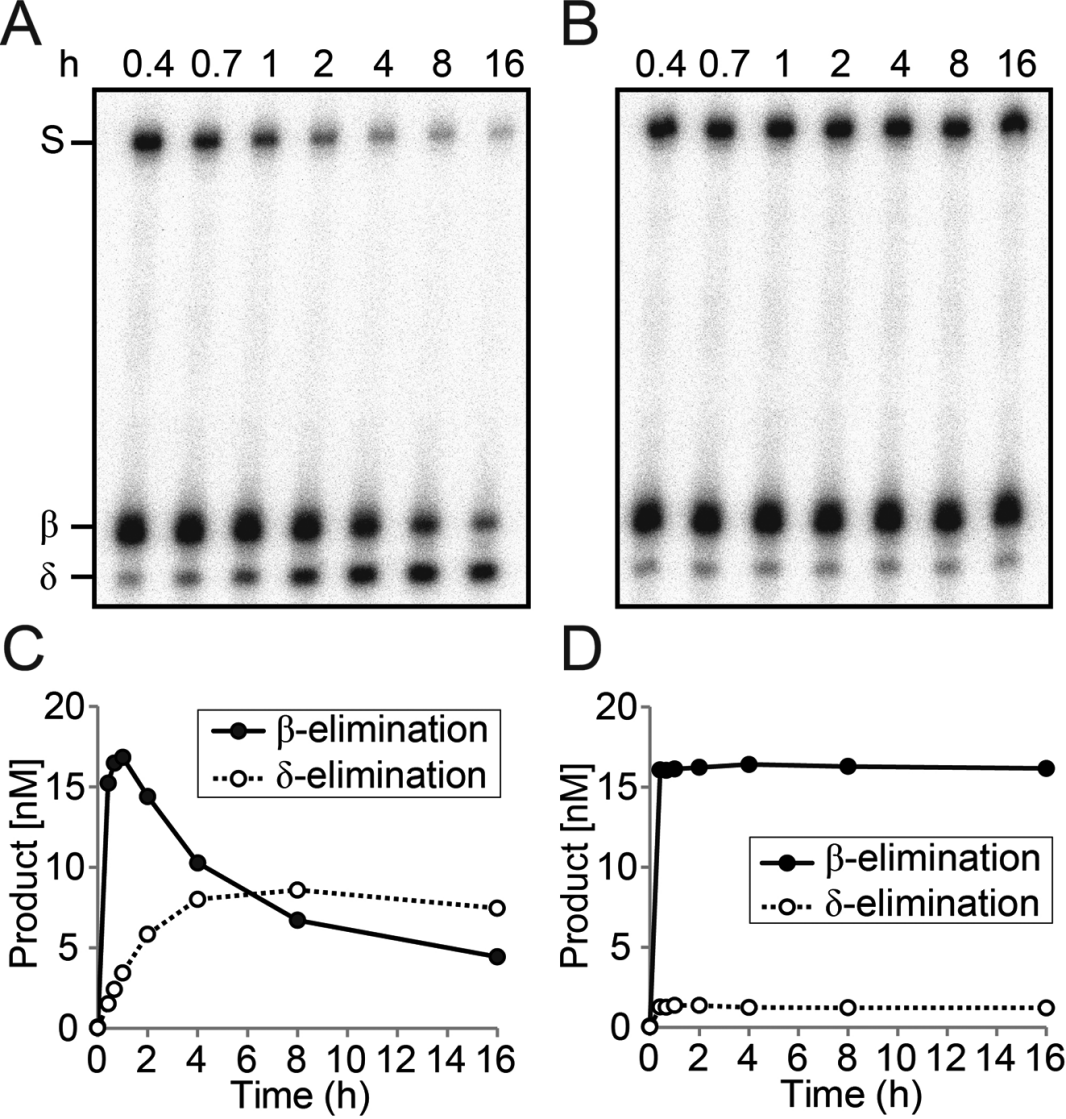
Enzyme-dependent δ-elimination process during 5mC excision. (A and B) Radiolabeled 35-mer oligonucleotide duplex containing 5mC at position 18 (25 nM) was reacted with purified MBP-DMEΔ (100 nM) at 37°C for 25 min. Reactions were separated and paused on ice (A) or heat-inactivated at 65°C (B) for 15 min, and then resumed at 37°C for additional 15.6 h. Reactions were terminated at indicated time points and separated on a polyacrylamide gel. (C and D) Reaction products from the experiments in (A and B) were quantitated and plotted over time.

### Cloning, expression and purification of *Arabidopsis* APE1L, APE2 and ARP

Full-length sequences of *APE1L*, *APE2* and *ARP* were obtained by reverse transcriptase-polymerase chain reaction (RT-PCR) from the whole plant of *Arabidopsis* Col-0. To generate the *APE1L* expression construct, the *APE1L* sequence was PCR-amplified using primers DG326 and DG327 with *Bam* HI and *Sal* I restriction sites, respectively (Supplementary Table S2). After digestion with *Bam* HI and *Sal* I, the PCR-amplified fragment was cloned at the corresponding sites of the pLM302 vector (Center for Structural Biology, Vanderbilt University) to create the pLM302-APE1L. The constructs pLM302-APE2 and pLM302-ARP were generated using primer pairs DG328 and DG329 for *APE2*, and DG160 and DG161 for *ARP*, respectively, by essentially following the same procedures as for *APE1L*. Bacterial expression and purification of APE1L, APE2 and ARP proteins are described in detail in Supplementary Information.

### AP endonuclease assay on AP sites

Oligonucleotides used in this experiment were synthesized from Integrated DNA Technologies (Supplementary Table S1). Forty pmols of 35-nt-long oligonucleotide containing a tetrahydrofuran (THF) at position 18 (F35[AP]) was radiolabeled with [γ-^32^P]ATP using T4 Polynucleotide Kinase (Takara) at 37°C for 1.5 h and annealed to the complementary strand to generate the AP site analog substrate. Oligonucleotide substrate (25 nM) was incubated with 5 nM each of MBP-APE1L, -APE2 and -ARP in the AP endonuclease reaction buffer (10 mM Tris-HCl, pH 7.4, 50 mM NaCl, 200 μg/ml BSA, 2.5 mM MgCl_2_, 0.5 mM DTT) at 37°C for 30 min. As a reaction control, the same oligonucleotide was reacted with 0.5 unit of hAPE1 (NEB). For kinetics analysis, various amounts of radiolabeled oligonucleotide substrate (0–100 nM) containing a THF was incubated with 5 nM MBP-ARP at 37°C for 4 min. Reaction was terminated by adding 100 mM NaOH and boiling for 10 min. Reactions were denatured and separated on a 15% denaturing polyacrylamide gel. The gel was exposed to a phosphorimager screen (Fujifilm) and the radioactivity was measured using the Fujifilm BAS-5000 phosphorimager.

### 3′ End cleaning assay

Radiolabeled 35-nt-long oligonucleotide containing 5mC at position 18 (F35[5mC]) was annealed to the complementary strand to prepare methylated DNA substrate. A total of 25 nM radiolabeled methylated oligonucleotide duplex was incubated with 100 nM MBP-DMEΔ in the glycosylase reaction buffer for 1 h at 37°C. In the same reaction tube, 5 nM each of MBP-APE1L, -APE2 and -ARP was added and reaction was performed at 37°C for 20 min in the presence of 2.5 mM MgCl_2_.

### *In vitro* nucleotide incorporation assay

A total of 25 nM radiolabeled oligonucleotide substrate containing 5mC was reacted with 100 nM MBP-DMEΔ and further reacted with 5 nM each of MBP-APE1L, -APE2 and -ARP at 37°C for 20 min. Following heat-inactivation at 65°C for 15 min, the reaction was subjected to nucleotide incorporation with 0.1 mM dCTP (Supplementary Figure S1) or 0.025 mM Cy3-dCTP (Figure 4) using 5 units of Klenow fragment (3′→5′ exo-) (NEB) at 25°C for 25 min.

**Figure 3.**
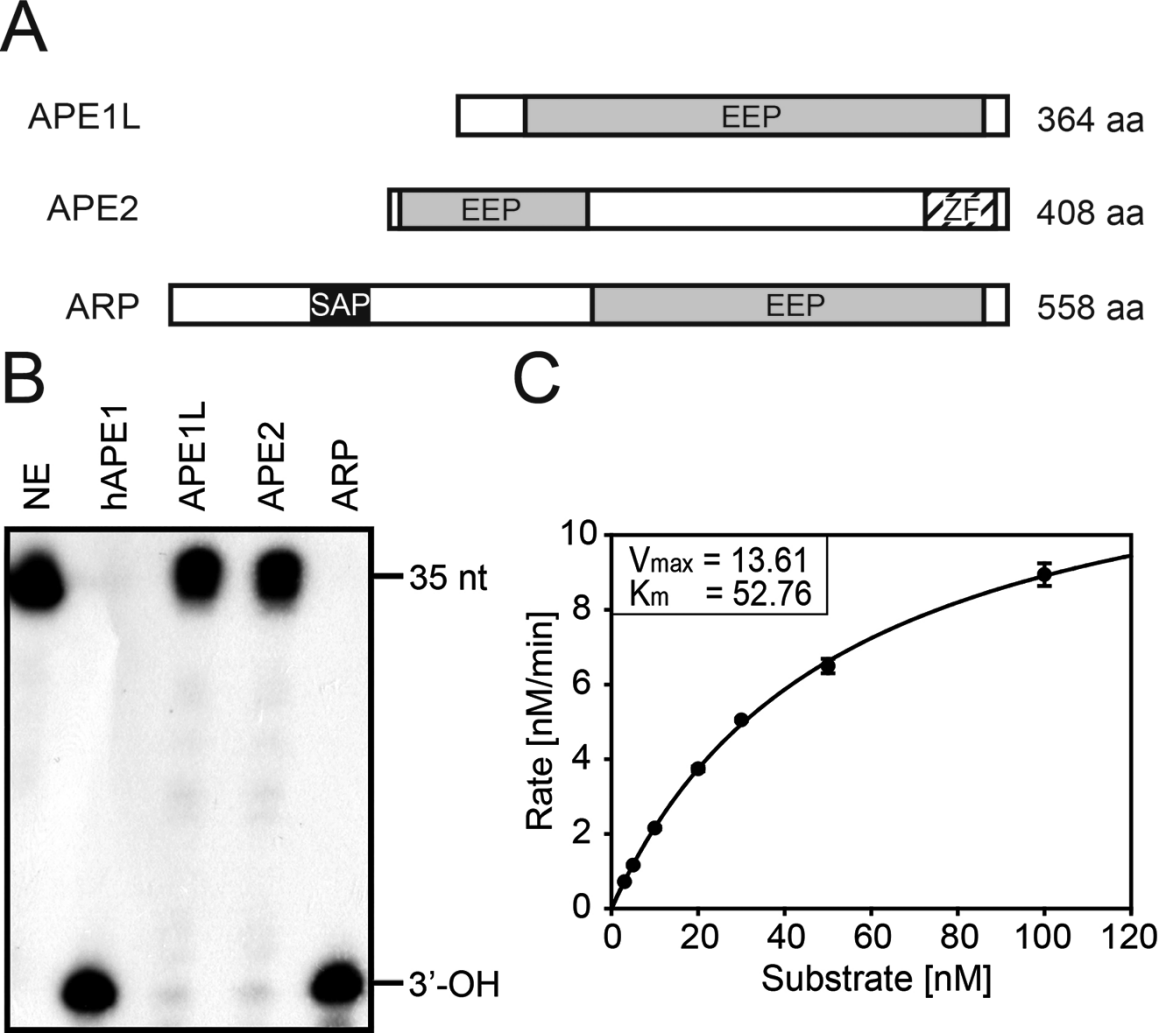
AP site incision assay of APE1L, APE2 and ARP. (A) Schematic representation of APE1L, APE2 and ARP proteins. EEP, endonuclease-exonuclease-phosphatase; SAP, SAF-A/B, Acinus and PIAS; ZF, GRF-type zinc finger motif. (B) AP endonuclease activity on the AP site. Radiolabeled 35-nt double-stranded DNA containing a THF, an AP site analog, at position 18 (F35[AP]) was used as a substrate for AP endonuclease assay. Reactions were done with 5 nM each of MBP-APE1L, -APE2 and -ARP at 37°C for 30 min. As a reaction control, 0.5 unit of hAPE1 was used. AP endonuclease reaction product (17-nt with 3′-OH) is indicated at the right of the panel. NE, no enzyme control. (C) Kinetics analysis of *Arabidopsis* AP endonucleases. The incision activity of ARP on AP site was measured by reacting purified MBP-ARP (5 nM) with varying concentrations of substrate (0–100 nM) at 37°C for 4 min. Error bars represent standard deviations from three independent experiments.

**Figure 4.**
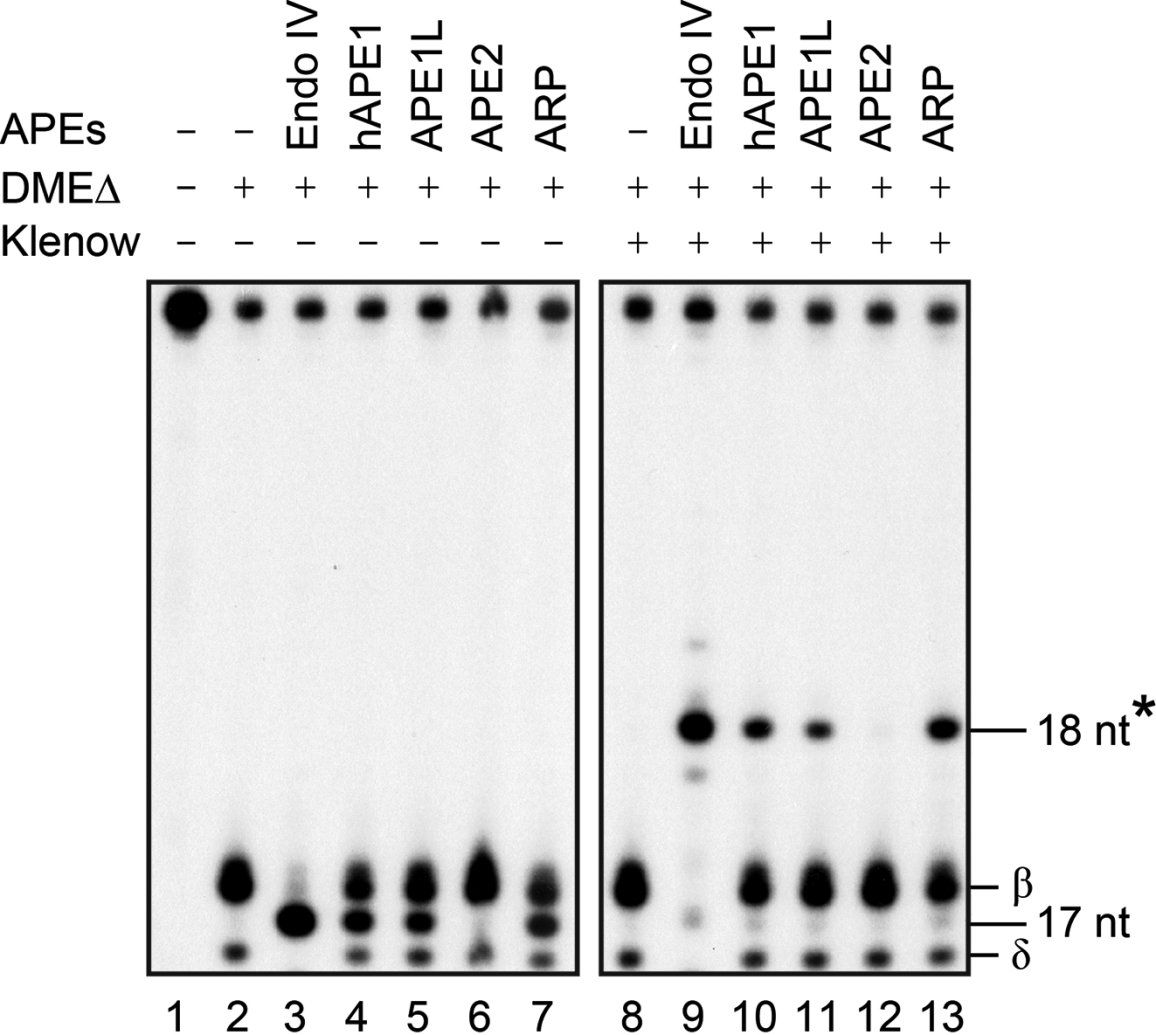
Reconstitution of DNA demethylation with *Arabidopsis* AP endonucleases *in vitro*. Radiolabeled 35-mer oligonucleotides containing 5mC were reacted with MBP-DMEΔ (lane 2), and the reaction product was further incubated with each purified *Arabidopsis* AP endonuclease in the presence of 2.5 mM MgCl_2_ (lanes 5–7). Subsequent Cy3-dCTP incorporation (denoted with an asterisk) was achieved by Klenow DNA polymerase to fill the gap generated by AP endonuclease (lanes 11–13). *E. coli* Endonuclease IV (lanes 3 and 9) and human hAPE1 (lanes 4 and 10) were done as controls. The 3′ end-processed (17-nt) and a cytidine-incorporated fragments (18-nt) were indicated relative to MBP-DMEΔ-treated products (β and δ) to the right of the panel. Endo IV, Endonuclease IV.

### 3′ Phosphatase assay

The 17-mer oligonucleotide with a 3′-phosphate was 5′-end-labeled with [γ-^32^P]ATP using T4 Polynucleotide Kinase (3′ phosphatase minus) (NEB). This radiolabeled upstream oligonucleotide and another 17-mer downstream oligonucleotide with a 5′-phosphate were annealed together to the 35-mer complementary strand to produce DNA substrate with a single nucleotide gap. A total of 25 nM DNA substrate was incubated with 5 nM each of AP endonucleases in the AP endonuclease reaction buffer at 37°C for 1 h. For kinetics analysis, 5 nM MBP-ARP was incubated with 25 nM radiolabeled oligonucleotide duplex in a time course manner (2, 5, 10, 30, 60, 120, 240, 480 and 960 min).

### Bacterial cell toxicity assay

For simultaneous expression of two different proteins in *Escherichia coli*, the pYOON01 vector was developed by reconstituting the pMAL-c2X and pSTV28 vectors to contain the p15a origin, the chloramphenicol resistance gene and a *tac* promoter (Supplementary Figure S2A). Each *APE1L*, *APE2* and *ARP* gene was cloned into the *Bam* HI and *Sal* I sites of the pYOON01 vector to produce a fusion with an N-terminal 6×His-MBP. Each construct was transformed into RPC501 strains (24) along with the pMAL-c2X or pMAL-c2X-DMEΔN677ΔIDR1::lnk (25). Fresh colonies were picked up and grown at 37°C overnight. The culture was serially diluted and spotted on LB/Amp plates with 0–125 μM isopropyl β-D-1-thiogalactopyranoside (IPTG). The plates were incubated at 26°C for 24–30 h.

## RESULTS

### DME and ROS1 5mC DNA glycosylases generate a 3′-PUA as a primary blocking intermediate in early 5mC excision

The first step of BER involves the base removal by DNA glycosylase, which cleaves an N-glycosidic bond between the base and a deoxyribose sugar creating an AP or abasic site (Figure 1A). Depending on the mode of base excision and the ability to execute strand cleavage, DNA glycosylases can be categorized into monofunctional and bifunctional enzymes (26). After base excision, bifunctional DNA glycosylases catalyze the scission of the sugar phosphate backbone leaving the 3′-PUA by the process called β-elimination, and further δ-elimination process generates a 3′-phosphate (Figure 1A). Such β- and δ-elimination products need to be immediately cleaned up to provide a 3′-OH for subsequent polymerization.

Previous studies showed that DME/ROS1 family 5mC DNA glycosylases catalyze both 5mC excision and a strand cleavage according to bifunctional mechanism, producing 3′-PUA and 3′-phosphate by β- and δ-elimination, respectively (6,7,9,18,19). To carefully monitor the changes in relative abundance of β- and δ-elimination processes, we performed kinetic studies on 5mC excision by DME and ROS1. When reacted with a 35-mer duplex oligonucleotide substrate that contained a 5mC residue at position 18 in the 5′-end-labeled strand, both DME and ROS1 produced 3′-PUA via β-elimination as a primary 5mC excision intermediate at early stages (<5 min) (Figure 1B and C). As the reaction proceeded, 3′-phosphate started to appear but its amount was significantly lower than 3′-PUA (Figure 1B). Prolonged reaction generated more 3′-phosphate while the amount of 3′-PUA gradually decreased, and after the reaction reached near completion, the 3′-phosphate became predominant (Figure 2A and C), implying that 3′-phosphate was the final 5mC excision product via δ-elimination of 3′-PUA.

These observations suggest that β-elimination predominantly occurs at early reaction stages and δ-elimination follows as a relatively slow process. In living cells, this may pose a serious problem to the DNA strands on which DNA demethylation takes place, particularly when a strand gap remains open for a long time. Importantly, two distinct enzyme activities may be required to completely clear 3′-end blocking structures 5′ to the cleavage site because 3′-PUA and 3′-phosphate are predominantly produced at different time windows by β- and δ-elimination, respectively.

### Enzyme-dependent δ-elimination process following 5mC excision

To ensure that sequential β- and δ-elimination reactions are intrinsic to DME/ROS1 glycosylases, we tested whether the latter δ-elimination process is spontaneous or enzyme-dependent. We incubated MBP-DMEΔ with a 35-mer duplex oligonucleotide containing a 5mC residue in the middle of the 5′-end-labeled top strand. Twenty-five minutes after start, two separate reactions were either terminated at 65°C or stalled on ice, and then both returned to 37°C for additional 15.6 h. We compared the δ-elimination rates between the two experiments by measuring the amounts of 3′-phosphate produced after reincubation (Figure 2A–D). When the reaction was paused on ice for 15 min and then resumed, 3′-phosphate was continuously produced while the remaining 3′-PUA gradually disappeared (Figure 2A and C). By contrast, when DME was denatured by heating at 65°C for 15 min, production of 3′-phosphate was no longer observed after the reaction resumed (Figure 2B and D), suggesting that δ-elimination is an enzyme-dependent process. This result also implies that successive β- and δ-eliminations are intrinsically coupled processes.

We have shown that DME/ROS1 5mC glycosylases catalyzed successive β- and δ-elimination processes after 5mC excision, generating 3′-phosphate as an end product (Figure 1B and C). In mammalian cells, 3′-phosphate termini are converted to 3′-OH by polynucleotide kinase 3′ phosphatase (PNKP) (27) and the corresponding enzyme activities were also reported in plants (28,29). Most importantly, ZDP was proposed to play a central role in active DNA demethylation acting downstream of ROS1 (20). If a 3′-phosphate was the only end product of 5mC excision by DME/ROS1, ZDP alone would effectively process the lesion to produce 3′-OH. However, the extremely slow turnover rate of 3′-PUA to 3′-phosphate by DME/ROS1-mediated δ-elimination, and early formation of 3′-PUA as a major intermediate (Figure 1B) indicate that an additional mechanism(s) is required to process 3′-PUA. Thus, we focus on the activities of plant AP endonucleases that are possibly involved in processing base excision intermediates.

### *Arabidopsis* encodes three putative AP endonucleases APE1L, APE2 and ARP that belong to the endonuclease/exonuclease/phosphatase (EEP) superfamily

The *Arabidopsis* genome has three genes *APE1L*, *APE2* and *ARP* that are homologous to AP endonuclease genes from bacteria and animals (Figure 3A and Supplementary Figure S3). All three AP endonucleases have a common EEP domain that displays a significant homology to *E. coli* Exonuclease III and human AP endonucleases hAPE1 and hAPE2 (Supplementary Figure S3). The EEP domain comprises most of the protein sequence of APE1L. APE2 and ARP have an additional zinc-finger (ZF) domain and SAP DNA binding domain, respectively (Figure 3A). AP endonucleases are highly conserved among diverse species (30) and the structures of *E. coli* Xth and hAPE1 propose the catalytic mechanism (31,32). Most of the residues directly involved in catalysis are highly conserved in all proteins except APE2, in which some essential amino acids (i.e. Asp210 and Asn212 in hAPE1) are missing (Supplementary Figure S3).

Quantitative RT-PCR analysis showed that all three AP endonucleases were expressed in most of the tissues examined (Supplementary Figure S4). Expression of *APE2* and *ARP* was observed in both vegetative and reproductive organs. By contrast, the transcription level of *APE1L* was significantly high in developing siliques, whereas no expression was detected in roots (Supplementary Figure S4). This suggests that, unlike APE2 and ARP, APE1L may have tissue-specific functions in *Arabidopsis*.

### Three *Arabidopsis* AP endonucleases APE1L, APE2 and ARP possess differential AP site incision activities

So far, ARP is the only *Arabidopsis* AP endonuclease whose biochemical activity was characterized using purified protein or cell extract (33,34). Therefore, for comprehensive understanding of their biochemical characteristics related to BER and active DNA demethylation, we cloned all three putative AP endonuclease genes from *Arabidopsis* and prepared recombinant proteins from *E. coli* for *in vitro* assay (Supplementary Figure S5). Note that all *Arabidopsis* AP endonucleases were expressed with an N-terminal MBP fragment, and its removal had no effect on enzyme activity compared to MBP fusion but somewhat decreased the protein stability. Therefore, we present all biochemical data in this study using MBP attached proteins. For the experiments using MBP-free AP endonucleases, see Supplementary Figures S6–S7.

As a core component of BER, AP endonuclease plays an essential role in processing AP sites generated either spontaneously or by monofunctional DNA glycosylases (30,35). AP endonuclease catalyzes the incision of the DNA-sugar phosphate backbone at 5′ to the AP site to prime DNA repair synthesis. In order to see whether *Arabidopsis* AP endonucleases have the canonical AP site incision activity, we incubated each AP endonuclease with an end-labeled 35-mer oligonucleotide duplex containing a single AP site analog THF at position 18. Consistent with previous reports (33,34), purified ARP showed the incision activity at the AP site generating 3′-OH, whereas neither APE1L nor APE2 displayed such activity (Figure 3B). The product formation rate of *Arabidopsis* ARP (*V*_max_ = 13.61 nM/min, *K*_m_ = 52.76 nM) was comparable to other characterized AP endonucleases, such as human AP endonuclease 1 (hAPE1) and *E. coli* Exonuclease III (Figure 3C and Supplementary Table S3). This result implies that ARP is the primary AP endonuclease in *Arabidopsis* to incise AP sites in the BER pathway acting immediately downstream of monofunctional DNA glycosylases.

### APE1L and ARP process major 5mC excision intermediate 3′-PUA to generate 3′-OH

During the course of active DNA demethylation, DME and ROS1 sequentially produce 3′-PUA and 3′-phosphate through β- and δ-elimination reactions, respectively (Figure 1B), but ZDP is known to have activity to process only the 3′-phosphate (20). In living cells, the persistence of 3′-PUA and 3′-phosphate in DNA is detrimental because they are part of single-strand break (SSB) damage serving as harmful blocking lesions in DNA replication and transcription (22). To test whether *Arabidopsis* AP endonucleases have activity to process a 3′-PUA and/or 3′-phosphate acting downstream of DME-catalyzed DNA demethylation, we prepared a radiolabeled oligonucleotide duplex with 5mC on the top strand. DNA substrate was first reacted with DME for 1 h and then heat-inactivated to prevent additional enzymatic base excision. The 1 h DME reaction products, in which a 3′-PUA was predominant and a 3′-phosphate was minor (lane 2 in Figure 4), were further reacted with APE1L, APE2 or ARP. Similarly to hAPE1 (lane 4 in Figure 4), *Arabidopsis* APE1L and ARP catalyzed the conversion of 3′-PUA to 3′-OH (lanes 5 and 7 in Figure 4), whereas APE2 displayed no discernable activity (lane 6 in Figure 4). The kinetics analysis of APE1L and ARP for 3′-phosphodiesterase activity revealed that the relative processing efficiency of ARP was ∼1.8-fold higher than APE1L (Supplementary Figure S8). All AP endonucleases showed no activity in the presence of EDTA (2 mM), indicating that these enzymes require Mg^2+^ for catalysis (Supplementary Figure S9).

These results suggest that APE1L and ARP are capable of removing 3′-PUA, allowing nucleotide extension by DNA polymerase. Interestingly, APE1L has significant activity for 5mC excision intermediates but not for an AP site analog THF (Figure 3B). This suggests that APE1L functions downstream of bifunctional DNA glycosylases, particularly in the DNA demethylation pathway, and that the 3′-end trimming process requires a distinct mechanism different from that of AP site incision.

### Reconstitution of DNA repair synthesis following 5mC excision

We expect that after 5mC excision by the DME/ROS1 family of proteins, subsequent BER enzymes participate in the DNA demethylation process by incorporating unmethylated cytidine in place of excised 5mC. We reconstituted the *in vitro* BER-mediated DNA demethylation pathway by showing the replacement of 5mC with unmethylated cytidine after DME base excision. When the radiolabeled oligonucleotide containing 5mC was reacted with DME for 1 h, 3′-PUA and 3′-phosphate were produced via β- and δ-elimination processes, respectively (lane 2 in Figure 4). Cy3-dCTP was incorporated at the site of 5mC excision by Klenow (3′→5′ exo-) fragment. Because incorporation of regular dCTP was hardly distinguishable from the spot corresponding to the 3′-PUA on an acrylamide gel (Supplementary Figure S1), we used Cy3-dCTP for better separation due to its high molecular weight. We observed that Cy3-dCTP was successfully inserted in the gap after the treatment of DME reaction products with APE1L or ARP (lanes 11 and 13 in Figure 4), but no extension took place when treated with APE2 (lane 12 in Figure 4). This demonstrates that both APE1L and ARP, but not APE2, are able to convert the β-elimination product 3′-PUA to 3′-OH allowing cytidine nucleotide incorporation after 5mC excision by DME.

### 3′ Phosphatase activity of ARP

**Figure 5.**
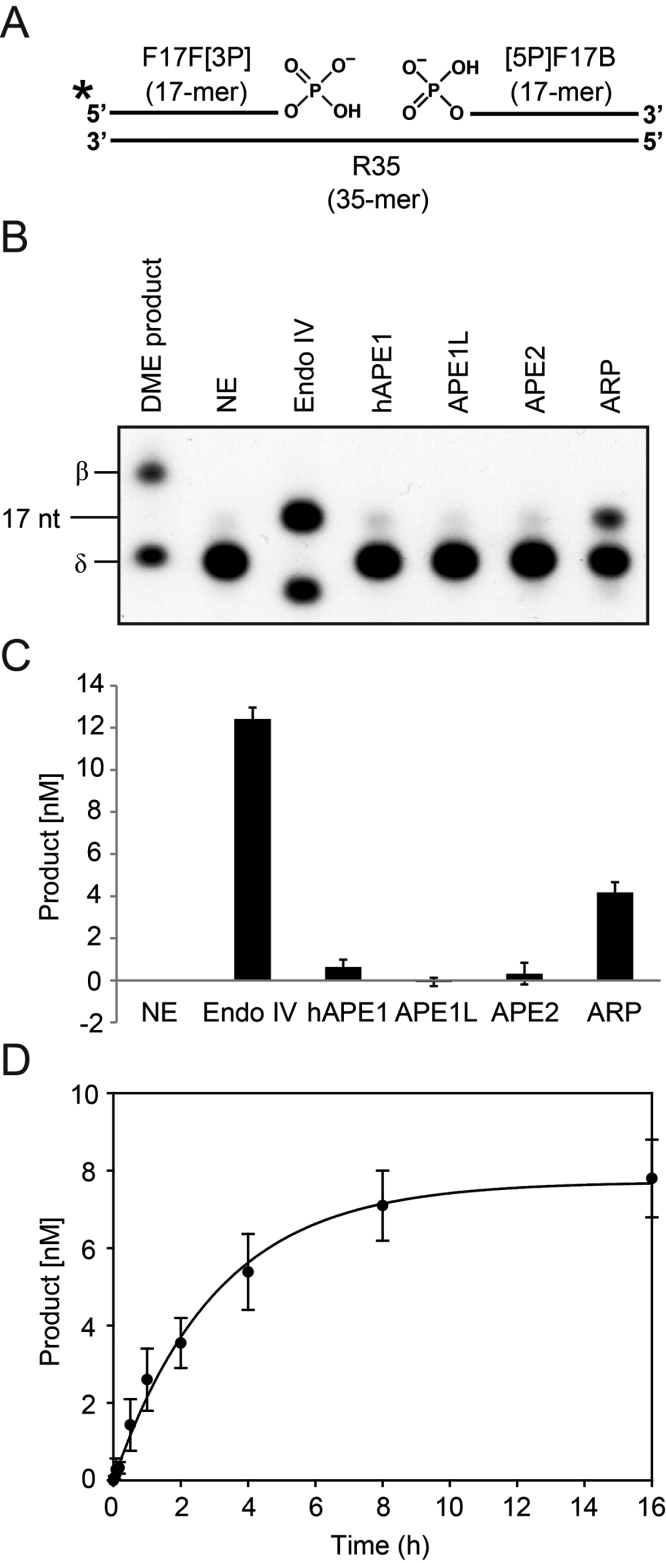
The 3′ phosphatase activity of *Arabidopsis* AP endonucleases. (A) Structure of 35-mer oligonucleotide duplex that mimics DME-catalyzed δ-elimination product for 3′ phosphatase assay. The radiolabeled upstream 17-mer oligonucleotide with a 3′-phosphate (F17F[3P]) and the downstream 17-mer with a 5′-phosphate ([5P]F17B) are annealed together to the complementary 35-mer strand (R35) to produce DNA substrate with a 1-nt gap in the middle. (B) The 3′ phosphatase activity of *Arabidopsis* AP endonuclease. DNA substrate depicted in (A) was reacted with purified MBP-APE1L, -APE2 or -ARP at 37°C for 1 h. Only MBP-ARP protein converted a δ-elimination product analog to 3′-OH, like *E. coli* Endonuclease IV (Endo IV). Either MBP-APE1L or -APE2 did not catalyze such conversion, like human APE1 (hAPE1). Methylated DNA substrate used for DNA glycosylase assay was reacted with MBP-DMEΔ and loaded alongside for size comparison. NE, no enzyme control. (C) Relative 3′ phosphatase activities of *Arabidopsis* AP endonucleases on a δ-elimination product. The amounts of 3′ phosphatase reaction products were measured by phosphorimager. (D) Kinetics analysis of ARP 3′ phosphatase activity. The above DNA substrate was reacted with MBP-ARP (5 nM) at 37°C in a time-course manner. The amounts of 3′ phosphatase reaction products at each time point were measured and plotted over time. Error bars represent standard deviations from three independent experiments (C and D).

We showed that DME catalyzed the β-elimination first to generate a 3′-PUA and a subsequent enzyme-assisted δ-elimination process produced a 3′-phosphate. This implies that an efficient removal of 3′-phosphate is a critical step toward nucleotide extension after 5mC excision. Since the 3′ phosphatase activity of *E. coli* Endonuclease IV was previously reported (36), we investigated whether *Arabidopsis* AP endonucleases are also capable of processing a 3′-phosphate produced by δ-elimination. Flanking a 1-nt central gap, both a radioactively 5′ end-labeled 17-nt oligonucleotide with a 3′-phosphate and an unlabeled 17-nt oligonucleotide with a 5′-phosphate were annealed to the complementary strand to prepare a 35-nt oligonucleotide duplex mimicking a δ-elimination product (Figure 5A). This substrate was reacted with each *Arabidopsis* AP endonuclease. As shown in Figure 5B, APE1L or APE2 did not convert the 3′-phosphate of δ-elimination product to 3′-OH. By contrast, ARP displayed significant 3′ phosphatase activity to process the 3′-phosphate generating 3′-OH, albeit relatively lower than that of *E. coli* Endonuclease IV (Figure 5B and C). The kinetics study showed that ARP processed the 3′-phosphate at a slow rate reaching its plateau after 8 h of reaction (Figure 5D). Despite its low activity *in vitro*, this result strongly suggests that ARP itself is capable of processing both DME-catalyzed 5mC excision intermediates 3′-PUA and 3′-phosphate with no support from other enzymes, such as a 3′ phosphatase.

### DNA binding of APE1L, APE2 and ARP

Several studies report that some AP endonucleases have additional functions besides the AP site processing, which include transcription factor stimulation and checkpoint signaling (33,37,38). For recruiting other proteins or modulating their activities, AP endonuclease should have a strong affinity to DNA where the events occur regardless of enzyme activity. Thus, we examined DNA binding of *Arabidopsis* AP endonucleases using the electrophoretic mobility shift assay (Supplementary Figure S10). All *Arabidopsis* AP endonucleases were found to bind DNA substrate containing THF or 5mC. Interestingly, even enzymatically inactive APE2 displayed strong affinity to DNA, suggesting that APE2 DNA binding is independent of enzyme activity. Also, considering methylated DNA is not a direct substrate of AP endonuclease, all *Arabidopsis* AP endonucleases appear to have non-specific DNA binding properties. This suggests that *Arabidopsis* AP endonucleases, including biochemically inactive APE2, may have some unknown biological functions.

### Bacterial expression of *APE1L* and *ARP* reduces DME-dependent 5mC excision toxicity

**Figure 6.**
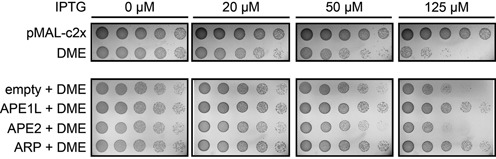
Complementation analysis with *Arabidopsis* AP endonucleases in AP endonuclease-deficient *E. coli*. Serial dilution assay to measure the cytotoxicity of *DME* expression in AP endonuclease-deficient RPC501 (*xth- nfo-*) strains with increasing amounts of IPTG (0–125 μM). The growth rate of RPC501 decreased as *DME* expression increased due to excessive 5mC excision. Expression of *APE1L* or *ARP* helped RPC501 strains to maintain cell growth by decreasing DME-induced cytotoxicity. *APE2* expression did not restore normal growth rates.

We previously showed that expression of *DME* was highly toxic to cytosine DNA methylation-proficient *E. coli* strains (*dcm*+), whereas little toxicity was observed in DNA methylation-deficient strains (*dcm*-) (7). Such DME-dependent toxicity was so prominent in AP endonuclease-deficient strain RPC501 (*xth*- *nfo*-) (24) that *E. coli* could not tolerate expression of *DME*, even at low levels (7). These findings strongly suggest that DME specifically recognizes 5mC as a primary substrate *in vivo* and that bacterial AP endonuclease activity is required to process harmful lesions formed during the course of 5mC excision. To test whether *APE1L*, *APE2* or *ARP* could complement mutations of their functional counterparts in bacteria, we simultaneously expressed *DME* and each of *Arabidopsis* AP endonucleases in RPC501 strains (Supplementary Figure S2). As shown in Figure 6, the growth of RPC501 cells was significantly reduced as *DME* expression increased. However, we observed that expression of *APE1L* or *ARP* profoundly recovered the growth rate of *DME* expressing RPC501 cells, whereas expression of the empty vector control or APE2 had little effect on cell growth (Figure 6). These results indicate that APE1L and ARP, but not APE2, are able to trim in a timely manner the 3′-end structure at a nick formed by DME-catalyzed 5mC excision, and strongly support our *in vitro* AP endonuclease activity (Figure 4). Notably, expression of *ZDP* could not relieve the toxic effect caused by *DME* expression (Supplementary Figure S2C), suggesting that ZDP alone is not sufficient to process 5mC excision lesions.

### Functional analysis of *Arabidopsis* AP endonuclease mutants

It was previously shown that AP endonuclease mutants in *Arabidopsis* have several defects in embryo and seed development (39). To understand *in vivo* functions of *Arabidopsis* AP endonucleases and 3′ phosphatase ZDP, double heterozygous F_1_ plants were generated from crosses between homozygous *zdp-2* and either one of *ape1l-1*, *ape2-1* or *arp-1* plant. The F_1_ plants were self-pollinated to generate F_2_ progenies, which were then genotyped for their allelic composition (Supplementary Table S4). We were able to obtain double homozygous mutants of *ape2-1 zdp-2* or *arp-1 zdp-2*, but failed to retrieve *ape1l-1 zdp-2* mutants. This suggests that the absence of ZDP cannot be compensated by APE2 or ARP. Every single or double mutant did not display any abnormal phenotype. In addition, we could not recover *ape1l-1 ape2-1* homozygous mutants, indicating that either APE1L or APE2 is required for normal growth and development in *Arabidopsis* even though APE2 appeared to have no biochemical activity.

## DISCUSSION

### DNA demethylation requires DNA repair machineries after 5mC removal

In plants, the family of 5mC DNA glycosylases, also known as the DME/ROS1 family proteins, are responsible for excision of 5mC at target DNA sequences for gene activation (7,17,40,41). During the course of 5mC excision, these 5mC DNA glycosylases produce 3′-PUA and 3′-phosphate by successive β- and δ-elimination processes, respectively (Figure 1A and B). As blocking lesions for subsequent nucleotide extension, these 3′ structures are to be immediately processed to form 3′-OH, and two enzyme activities are likely engaged. One involves 3′ phosphatase activity, for which ZDP was recently proposed to remove 3′-phosphate after 5mC excision (20). The other may employ 3′ phosphodiesterase or 3′→5′ exonuclease activity provided by AP endonucleases. It is worth noting that DME and ROS1 have a significantly slow turnover rate for base excision compared to other conventional DNA glycosylases, and thus the production of 3′-PUA and 3′-phosphate takes a significant amount of time for reaction completion (Figure 2A). More importantly, the end product formed by DME/ROS1 proteins is a 3′-phosphate, and the responsible δ-elimination reaction is an enzyme-dependent and extremely slow process (Figure 2A–D). This implies that 3′ phosphatase activity of ZDP by itself cannot process 3′-phosphate immediately upon 5mC excision, which should bring about a critical damage on DNA due to persisting SSBs and 3′ blocking ends. Thus, in order for living cells to maintain genome integrity during DNA demethylation, there must be some other enzyme activities for the removal of 3′-PUA and/or 3′-phosphate shortly after 5mC excision, and as discussed below in detail, our study strongly suggests that AP endonucleases are more apt to perform such tasks. By contrast, mammalian systems might suffer a minimal degree of DNA damage during DNA demethylation as encountered in plants, because one of the enzymes that are primarily involved in the pathway is TDG (11,42). As a monofunctional DNA glycosylase, TDG excises TET-mediated 5mC oxidation derivatives, such as 5fC and 5caC, without forming a SSB, and the resulting AP site will be instantly processed by downstream AP endonucleases (for review see (43)). It is noteworthy that there also exists another branch of BER pathway in humans, where PNKP processes 3′-phosphate produced by NEIL-1 and NEIL-2 DNA glycosylases in an AP endonuclease-independent manner (44).

### *Arabidopsis* AP endonucleases are capable of processing diverse base excision intermediates

*Arabidopsis* has three AP endonucleases APE1L, APE2 and ARP (Figure 3A) but only ARP has been characterized for its biochemical activity (33,34). *Arabidopsis* ARP was reported to have an AP site incision activity toward acid-depurinated DNA (33), and to play an essential role in progression of short- and long-patch BER (34). However, the *in vitro* activities of other AP endonucleases, such as APE1L and APE2, were not comparatively examined under the same reaction conditions. The comprehensive analysis of three AP endonucleases demonstrate that only ARP has the AP site incision activity (Figure 3B), and both APE1L and ARP are capable of processing 3′-PUA to generate 3′-OH allowing subsequent nucleotide incorporation (Figure 4). By contrast, APE2 appears to have no biochemical activity for any substrate examined in this study. Notably, ARP has 3′ phosphatase activity like *E. coli* Endonuclease IV (Figure 5B and C) (36), which strongly suggests that ARP itself might be sufficient to process diverse base excision intermediates, such as an AP site, 3′-PUA and 3′-phosphate. In particular, the ability of ARP to process both 3′-PUA and 3′-phosphate suggests the possibility that ARP can effectively remove 5mC excision intermediates without the participation of another enzyme, such as ZDP 3′ phosphatase.

Recently, wheat APE1L (TaAPE1L) was isolated and its biochemical activities were examined *in vitro* (45). Besides AP site incision activity, TaAPE1L was shown to have 3′-phosphodiesterase, 3′-phosphatase and 3′→5′ exonuclease activities (45). Interestingly, unlike most other AP endonucleases, TaAPE1L was active in the presence of Mn^2+^, Co^2+^ and Fe^2+^ cations but inhibited by Mg^2+^ and Ca^2+^. However, our experiments revealed that unlike TaAPE1L, *Arabidopsis* APE1L did not show discernable AP site incision activity even in the presence of non-ionic detergent and MnCl_2_ or CoCl_2_ at lower temperature (23°C) (Supplementary Figure S11). This indicates that *Arabidopsis* APE1L and TaAPE1L have distinct functions and that *Arabidopsis* ARP requires a reaction condition different from that of TaAPE1L for AP site processing.

We also verified the *in vivo* functions of *Arabidopsis* AP endonucleases in the heterologous bacterial system. We previously reported that expression of *DME* was toxic to DNA methylation-proficient *E. coli* strain (*dcm+*), and the cytotoxicity was more exaggerated in AP endonuclease mutant RPC501 (*nfo- xth-*) due to excessive 5mC excision and concomitant SSB formation (7,25). We found that expression of *APE1L* or *ARP* along with *DME* significantly ameliorated DME-induced toxicity (Figure 6), indicating that these two AP endonucleases were able to compensate for the lack of endogenous bacterial AP endonuclease functions. However, *ZDP* expression was not helpful for cell survival, indicating that timely processing of DNA demethylation intermediate requires AP endonuclease rather than ZDP 3′ phosphatase activity.

### Distinct roles of AP endonucleases for plant development

Previous genetic studies on AP endonuclease mutants propose that at least one of intact *APE1L* and *APE2* genes is necessary for proper seed development in *Arabidopsis* (39). The same results were obtained from our genetic crosses among AP endonuclease mutants, in which no *ape1l ape2* double homozygous mutant was retrieved. This indicates a functional redundancy between *APE1L* and *APE2*, albeit *ARP* can be dispensable for reproductive development. This is quite surprising not only in that ARP was shown to process most harmful lesions that can be generated during the course of base excision, but also in the fact that APE2 appeared to have no catalytic activity considering its apparent lack of essential residues. However, we still cannot rule out the possibility that some enzymes are able to replace the function of ARP, and more importantly, APE2 has functions other than that of canonical AP endonuclease. Supporting this idea, several studies report that AP endonuclease plays a pivotal role for signal transduction and activation of transcription factors in diverse organisms. For example, *Xenopus* APE2 is known to activate checkpoint kinase 1 (Chk1) in response to oxidative stress using its Chk1-binding motif (37). Human APE1 also functions as a regulatory protein, stimulating the DNA binding activity of transcription factors Fos and Jun by a redox-dependent mechanism (38), and the similar effect was reported for *Arabidopsis* ARP (33). Interestingly, our seemingly paradoxical observations are somewhat reminiscent of the synthetic lethality observed in *Saccharomyces cerevisiae*, in which AP endonuclease-deficient *apn1 apn2* mutant strains display severe growth defects when combined with mutations of *TPP1* and *RAD1*/*RAD10* genes (46–48). Considering *TPP1* and *RAD1*/*RAD10* encode DNA 3′-phosphatase and 3′-flap endonuclease components, respectively, genetic studies in yeast imply that harmful AP sites can also be processed by DNA repair machineries other than AP endonuclease itself. Our findings of DNA binding activity of all AP endonucleases (Supplementary Figure S10) and the 3′→5′ exonuclease activity of APE1L (Supplementary Figure S11) strongly suggest that each *Arabidopsis* AP endonuclease may have some distinct functions, and in the light of the results of genetic studies in yeast, this might explain in part why APE1L or enzymatically inactive APE2 is still needed for normal growth and development in *Arabidopsis* and why we could not recover *ape1l zdp* double mutants from a genetic cross.

Considering proper regulation of DNA methylation is important for female gametophyte-specific gene imprinting (4,7,17), it is possible that such defects in reproductive development result from dysregulation of DNA demethylation because DME establishes central cell-specific hypomethylation at the target genes, such as *MEA* and *FIS2*, for transcriptional activation (4,7,17,41,49). Therefore, mutations in AP endonucleases are likely to block efficient DNA demethylation by DME in the central cell before fertilization, leading to gametophyte lethality. However, we cannot exclude the possibility that there is an unknown mechanism that allows DNA demethylation independently of AP endonuclease activity in the female gametophyte.

Another study proposes that ZDP 3′ phosphatase is essential for DNA demethylation and its mutation causes DNA hypermethylation and transcriptional gene silencing of a reporter gene (20). However, *zdp* mutants also do not exhibit any conspicuous developmental defects, albeit the cell extract from the mutant plant is impaired in the progression of DNA demethylation *in vitro* (20). This suggests the presence of ZDP-independent DNA demethylation pathway, and according to our data, AP endonucleases are likely to replace ZDP 3′ phosphatase function. We found that *ape2 zdp* or *arp zdp* double homozygous mutants were normal in growth and development, but the lack of *ape1l zdp* double mutant suggests a distinct function of APE1L for reproduction, which can be partly compensated by ZDP. It would be of much interest to see whether *APE1L* has a maternal effect in the presence of *zdp* mutation and is actually required for the establishment of female gametophyte-specific DNA demethylation and gene activation by DME.

### Model for the DNA demethylation pathway in *Arabidopsis*

**Figure 7.**
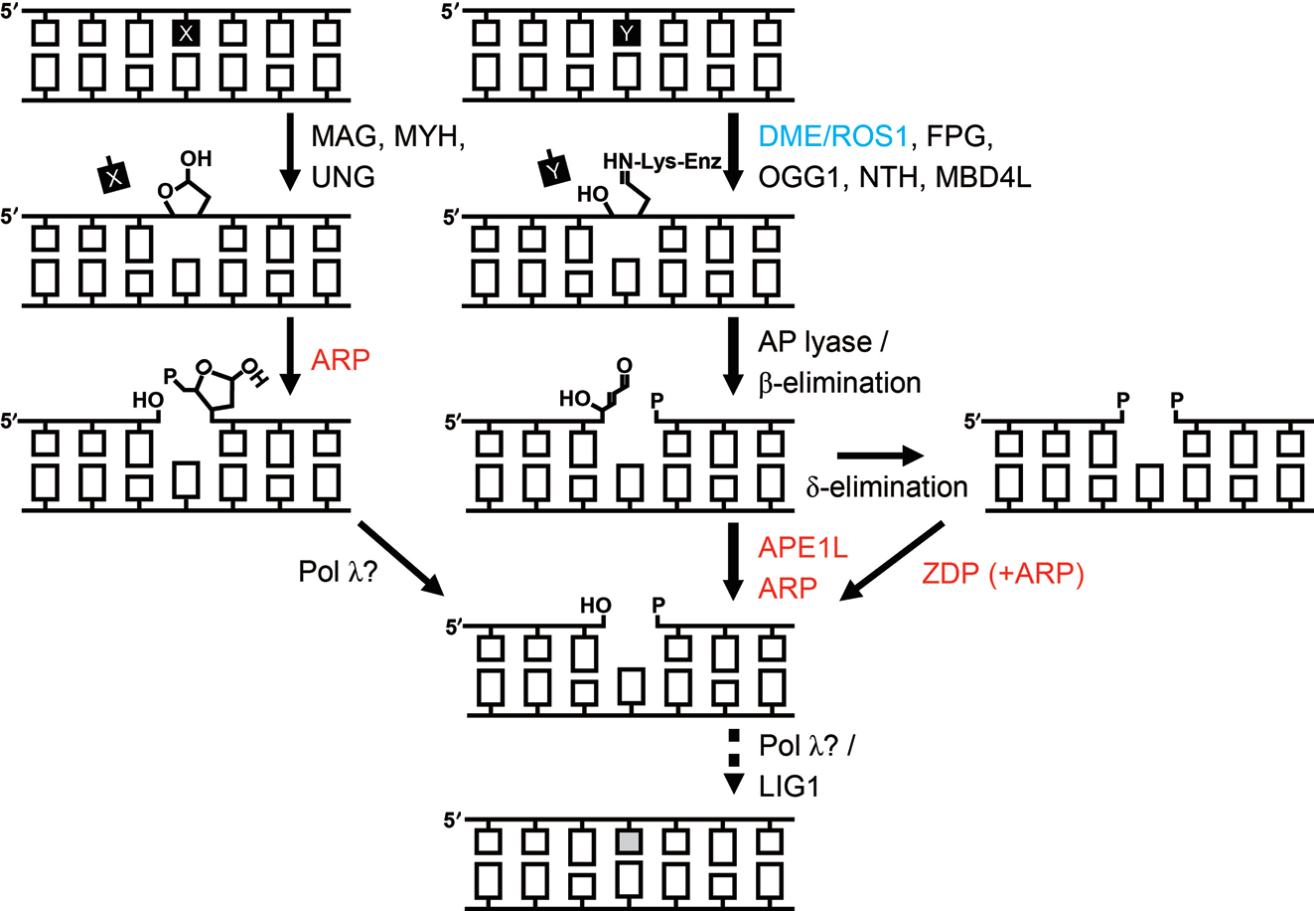
Model of active DNA demethylation and BER pathway in *Arabidopsis*. Various forms of bases are addressed by monofunctional or bifunctional DNA glycosylases. After base excision by monofunctional DNA glycosylases, the AP endonuclease ARP primarily catalyzes the cleavage of the phosphodiester bond, leaving a 3′-OH and 5′-dRP (shown in the left). The resulting 5′-dRP is presumably processed by DNA polymerase λ to generate 5′-phosphate at the nick. For bifunctional DNA glycosylases including the DME/ROS1 family of 5mC DNA glycosylases, base excision and a strand break simultaneously occur due to associated AP-lyase activity (shown in the middle). The resulting 3′-PUA is either directly processed by AP endonucleases APE1L and ARP, or further converted to 3′-phosphate via δ-elimination. This 3′-phosphate can be processed by ARP or ZDP 3′ phosphatase (shown in the right). Consequently, DNA polymerase λ and DNA ligase I (LIG1) will complete BER. The proposed pathway of active DNA demethylation in *Arabidopsis* is denoted by thick arrows in the middle. The basic properties of DNA glycosylases that have been identified in plants so far are listed in Supplementary Table S5.

Biochemical studies on *Arabidopsis* AP endonucleases in this study led us to propose a model of plant-specific BER and DNA demethylation pathways (Figure 7). A damaged, mismatched or modified (5mC in this case) base is recognized by monofunctional or bifunctional DNA glycosylases. Monofunctional DNA glycosylases, such as MAG, MYH and UNG, catalyze only the scission of an N-glycosidic bond between a base and a ribose sugar leaving an AP site (the pathway on the left in Figure 7), whereas bifunctional enzymes, such as FPG, OGG1, NTH, MBD4L and importantly, DME/ROS1 family DNA demethylases cleave the phosphodiester bond 3′ to the AP site, concomitantly with base removal using an intrinsic AP-lyase activity (the pathway in the middle in Figure 7). The primary base excision intermediates have abnormal 3′-end structures – 3′-PUA and 3′-phosphate generated by β- and δ-elimination processes, respectively. The 3′-PUA is removed by APE1L or ARP, and further progressed δ-elimination product 3′-phosphate can be processed by ZDP or ARP. According to our study, ARP is the only enzyme that is able to process all base excision intermediates including the AP site created by monofunctional DNA glycosylases or by spontaneous depurination. After the generation of 3′-OH, DNA polymerase will insert a nucleotide and DNA ligase seal the gap. It must be noted that DNA synthesis can take place either by a single nucleotide insertion (short-patch BER) or by nucleotide extension and strand displacement, generating a ‘flap’ structure (long-patch BER), and that different classes of DNA polymerases are employed in each pathway (50). The final ligation step of BER in *Arabidopsis* is likely done by DNA ligase I (LIG1) (34).

Currently, the mechanism of DNA synthesis after base excision in the plant cell is poorly understood. For BER in mammalian cells, DNA polymerase β is regarded as a major player catalyzing repair DNA synthesis, and at the same time, its associated 5′-dRPase activity processes the 5′-dRP to generate a 5′-phosphate for ligation. Plants do not appear to have any homologs of DNA polymerase β, but instead DNA polymerase λ is thought to perform the equivalent function (51–53). In addition, even though several reports have proposed the preference of short- or long-patch BER for repair of different lesions (34,53,54), it is still unclear whether plants prefer short- or long-patch BER for DNA demethylation. Since 5mC often occurs as a cluster in DNA to regulate gene expression and chromatin structure, it will be a daunting task if 5mC excision and its replacement with unmethylated cytidine take place one by one. Rather, the long-patch BER or nucleotide excision repair pathway may be a more efficient way to remove a stretch of methylated DNA strand at a time. Therefore, given the fact that active DNA demethylation utilizes many of DNA repair systems, it will be of great importance to investigate the repair machineries acting downstream of 5mC excision to better understand the molecular dynamics of DNA demethylation processes. This will also provide us with an evolutionary insight into how plants and animals have evolved distinct DNA demethylation systems for epigenetic gene regulation.

## SUPPLEMENTARY DATA

Supplementary Data are available at NAR Online.

SUPPLEMENTARY DATA
