# Supplementary material for: AP endonucleases process 5-methylcytosine excision intermediates during active DNA demethylation in Arabidopsis
Source: Nucleic Acids Res. 2014 Sep 16;42(18):11408–18. doi: 10.1093/nar/gku834 (PMC4191409; doi:10.1093/nar/gku834)
Supplement: SUPPLEMENTARY DATA [file supp_42_18_11408__index.html]

AP endonucleases process 5-methylcytosine excision intermediates during active DNA demethylation in Arabidopsis — AP endonucleases process 5-methylcytosine excision intermediates during active DNA demethylation in Arabidopsis — SUPPLEMENTARY DATA 

# AP endonucleases process 5-methylcytosine excision intermediates during active DNA demethylation in *Arabidopsis*

## SUPPLEMENTARY DATA

**Files in this Data Supplement:**

- SUPPLEMENTARY DATA
