# Supplementary material for: AP endonucleases process 5-methylcytosine excision intermediates during active DNA demethylation in Arabidopsis
Source: Nucleic Acids Res. 2014 Sep 16;42(18):11408–18. doi: 10.1093/nar/gku834 (PMC4191409; doi:10.1093/nar/gku834)
Supplement: SUPPLEMENTARY DATA [file supp_gku834_nar-01672-d-2014-File009.pdf]

AP Endonucleases Process 5-Methylcytosine Excision Intermediates during Active DNA Demethylation in *Arabidopsis*

Jiyoon Lee, Hosung Jang, Hosub Shin, Woo Lee Choi, Young Geun Mok, and Jin Hoe Huh*

SUPPLEMENTARY INFORMATION

Material and Methods

Expression and purification of APE1L, APE2 and ARP proteins

The pLM302-APE1L, -APE2, and -ARP vectors were transformed into *E. coli* Rosetta2 (DE3) strain (EMD Millipore). A single fresh colony was inoculated in 5 mL of LB medium containing kanamycin (50 μg/mL) and chloramphenicol (50 μg/mL). The culture was incubated at 37 °C overnight. An aliquot of overnight culture was inoculated into 2 L LB medium with the same antibiotics and incubated at 30 °C until OD_{600} reached 0.4. Expression was induced with 0.1 mM IPTG at 16 °C overnight with shaking. Cells were harvested by centrifugation and the pellet was resuspended in 30 mL of lysis buffer (50 mM Tris-HCl, pH 7.4, 100 mM NaCl, 10% glycerol, 0.1 mM dithiothreitol, 0.1 mM PMSF). The lysate was sonicated and clarified by centrifugation. The supernatant was collected and purified through the HisTrap FF column (GE Healthcare). Collected fractions were concentrated and stored in a storage buffer (20 mM Tris-HCl, pH 7.4, 100 mM NaCl, 10% glycerol, 0.1 mM DTT) at -80 °C until use.

RNA extraction and gene expression analysis

Total RNA was extracted from various tissues of *Arabidopsis* Col-0 plants using the TRIZOL reagent (Invitrogen). First-strand cDNA was synthesized from total RNA using Oligo(dT) primers with SuperScript II Reverse Transcriptase (Invitrogen). Primers used for quantitative RT-PCR analysis of *APE1L, APE2,* and *ARP* are listed in Supplementary Table S2.

Cloning of ZDP

The full-length ZDP cDNA was synthesized from total RNA of the whole plant of *Arabidopsis* Col-0 and the coding sequence was PCR-amplified from cDNA with primers DG1210 and

DG1211. The pYOON02 vector was made by modifying the MCS of the pYOON01 to contain *Eco* RI-*Kpn* I-*Xho* I-*Spe* I-*Sal* I using oligomers DG1335 and DG1336. The ZDP fragment was PCR-amplified using primers DG1215 and DG1211, and cloned into the pYOON02 at the sites of *Eco* RI and *Xho* I.

Table S1. List of oligonucleotides for biochemical assays.

name	oligonucleotide sequence
F35[AP]	5'- GTACTGTGTGATACTAT[THF]GAATTCAGTATGATCTG
F35[5mC]	5'- GTACTGTGTGATACTAT[5mC]GAATTCAGTATGATCTG
R35	5'- CAGATCATACTGAATTCGATAGTATCACACAGTAC
F30[5mC]	5'- CTGTGTGATACTAT[5mC]GAATTCAGTATGATC
R30	5'- GATCATACTGAATTCGATAGTATCACACAG
F17F[3P]	5'- GTACTGTGTGATACTAT[3Phos]
[5P]F17B	5'- [5Phos]GAATTCAGTATGATCTG

*THF, tetrahydrofuran; 5mC, 5-methylcytosine; Phos, phosphorylation

Table S2. List of primers for PCR amplification.

name	primer sequence (5'→3')
DG326	AATTGGATCCATGAAGCGATTCTTCAAGC
DG327	AATTGTCGACTTAGTTTGACACCTGGTTCTGTTC
DG328	AATTGGATCCATGGATCGATGTGAAGCTG
DG329	AATTGTCGACTTACTTGTCTCTGAATTTTGATG
DG160	AATTGGATCCATGAACAACGTTCTTCAGTTTGG
DG161	AATTGTCGACTCAGAGCTTGAGAATAAGGCCAATGG
DG1210	AATTGGATCCATGATTACAGTAGCCCCCTTCGTGTCT
DG1211	AATTCTCGAGCTAAGTCCCTGGCGATGTACTTGAGG
DG1215	AATTGAATTCATGATTACAGTAGCCCCCTTCGTGTCT
DG1335	AATTCGGTACCCTCGAGACTAGTG
DG1336	TCGACACTAGTCTCGAGGGTACCG

Table S3. Previous kinetics studies of AP endonucleases.

enzyme	substrate	k_{cat} (s^{-1})	K_m (nM)	k_{cat}/K_m	references
hAPE1	THF·G	1.80E+00	1.19E+01	1.51E-01	(1)
	THF·G	1.31E+01	1.07 E+01	1.22E+00	(2)
	THF·G	5.00E-01	1.90E+01	2.63E-02	(3)
	THF·G	3.37E+00	1.54E+02	2.19E-02	(4)
AtARP	THF·G	4.54E-02	5.28E+01	8.59E-04	reported in this paper
NApe	THF·G	3.10E-02	3.00E-01	1.03E-01	(5)
LMAP	THF·G	5.35E+00	2.00E+02	2.68E-02	(4)

Table S4. Genotypes of F_2 progeny from the self-cross of a double heterozygous AP endonuclease and *zdp* mutant plants.

F_1	F_2			
	APE1L	*APE2*	*ARP*	*ZDP*
APE1L$^{+/-}$ *APE2*$^{+/-}$	+/-	+/-	+/+	+/+
	-/-	+/-	+/+	+/+
APE1L$^{+/-}$ *ARP*$^{+/-}$	+/-	+/+	+/-	+/+
	-/-	+/+	+/-	+/+
APE2$^{+/-}$ *ARP*$^{+/-}$	+/+	+/-	+/-	+/+
	+/+	+/-	-/-	+/+
APE1L$^{+/-}$ *ZDP*$^{+/-}$	-/-	+/+	+/+	+/-
	+/-	+/+	+/+	-/-
APE2$^{+/-}$ *ZDP*$^{+/-}$	+/+	+/-	+/+	+/-
	+/+	+/-	+/+	-/-
	+/+	-/-	+/+	-/-
ARP$^{+/-}$ *ZDP*$^{+/-}$	+/+	+/+	+/-	+/-
	+/+	+/+	-/-	+/+
	+/+	+/+	-/-	-/-

Table S5. DNA glycosylases in *Arabidopsis thaliana.*

gene	locus	homolog	substrate[a]	AP lyase	references
MYH	At4g12740	MutY	**A**:8-oxoG	No	N/A
UNG	At3g18630	UDG	**U**:G	No	(6)
MAG	At3g12040	AAG, MPG, AlkA	**3-meA, 7-meG**	No	N/A
FPG1 (MMH1) FPG2 (MMH2)	At1g52500.2 (FPG1) At1g52500.1 (FPG2)	MutM	**8-oxoG**:C, **fapy**	Yes	(7-11)
OGG1	At1g21710	hOGG1	**8-oxoG**:C, **8-hG**:C	Yes	(12-15)
NTH1	At2g31450	Nth, Ntg1, Ntg2	**Tg, ox-Py**	Yes	(16)
NTH2	At1g05900	Nth, Ntg1, Ntg2	**Tg, ox-Py**	Yes	(16)
DME	At5g18090	-	**5mC**, **T**:G, **5hmC, 5caC**:G	Yes	(17-21)
ROS1	At2g36490	-	**5mC**, **T**:G, **5hmC, 5caC**:G	Yes	(20,22-24)
DML2	At3g10010	-	**5mC**, **T**:G	Yes	(19)
DML3	At4g34060	-	**5mC**, **T**:G, **5hmC, 5caC**:G	Yes	(19,21)
MBD4L	At3g63030	MBD4	**U**:G, **T**:G	Yes	(25)

[a] 3-meA, 3-methyladenine; 5-mC, 5-methylcytosine; 7-meG, 7-methylguanine; 8-oxoG, 8-oxo-guanine; fapy, formamidopyrimidine; ox-Py, oxidized pyrimidine; Tg, thymine glycol.

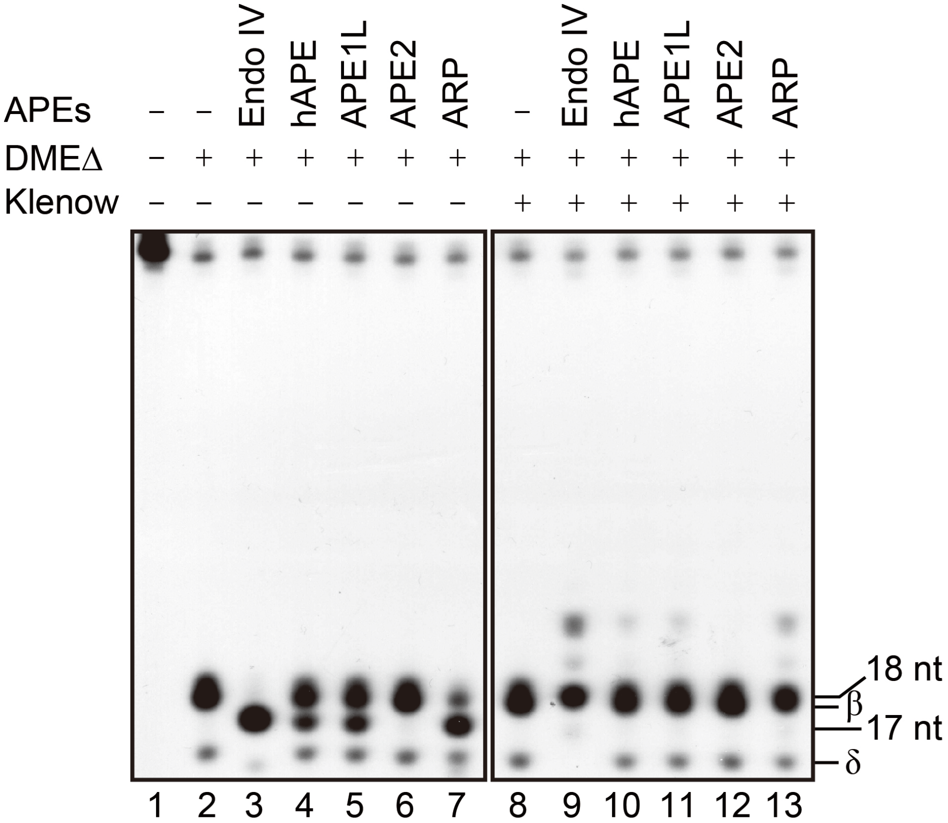

Figure S1. Reconstitution of DNA demethylation with *Arabidopsis* AP endonucleases *in vitro*.

Radiolabeled 35-mer oligonucleotides containing 5mC were reacted with DME (lane 2), and the reaction product was further incubated with each purified *Arabidopsis* AP endonuclease in the presence of 2.5 mM $MgCl_2$ (lanes 5-7). Subsequent dCTP incorporation was achieved by Klenow DNA polymerase (3'→5' exo-) to fill the gap generated by *Arabidopsis* AP endonucleases (lanes 11-13). *E. coli* Endonuclease IV (lanes 3 and 9) and human hAPE1 (lanes 4 and 10) were used as controls. The 3' end-processed (17-nt) and a cytidine-incorporated fragments (18-nt) were indicated relative to DME treated products (β, δ) to the right of the panel. Endo IV, Endonuclease IV.

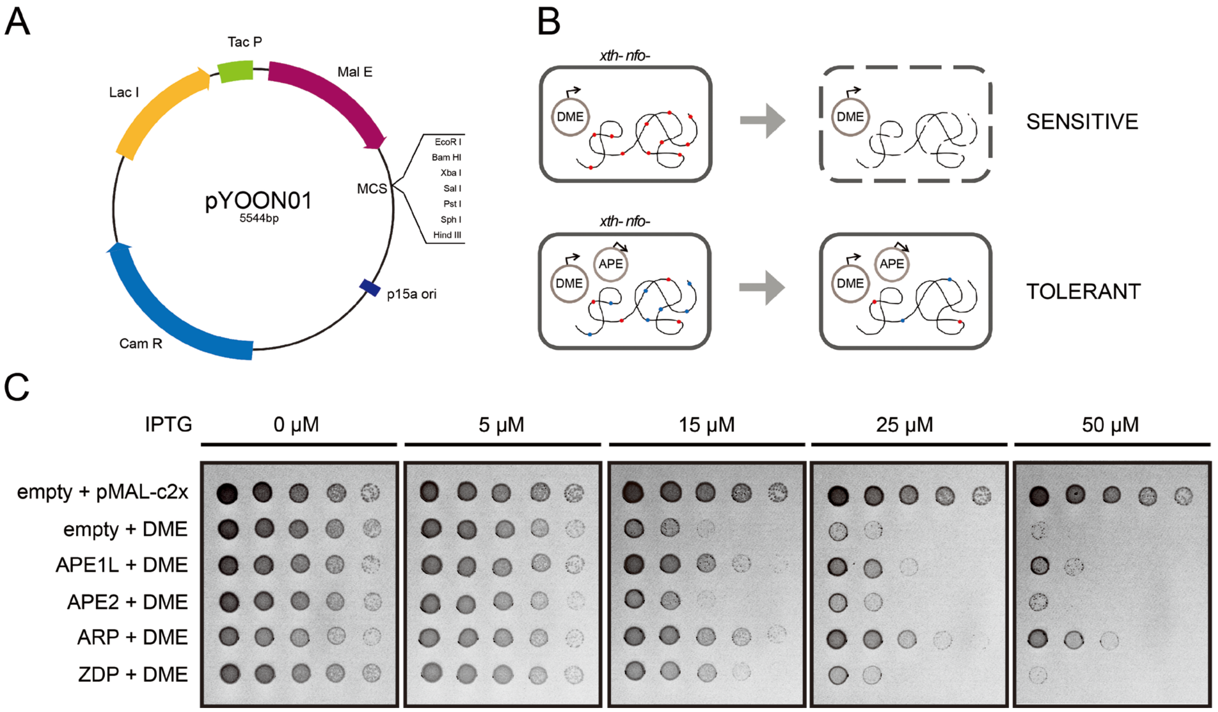

Figure S2. Complementation analysis with *Arabidopsis* AP endonucleases in AP endonuclease-deficient *E. coli*.

(A) A schematic diagram of the pYOON01 vector. The p15a origin and the chloramphenicol resistance gene (Cam R) are derived from the pSTV28 vector, and the *tac* promoter and the downstream *Mal E* gene are derived from the pMAL-c2X vector. (B) Scheme of toxicity test in *E. coli* strain RPC501 with AP endonuclease mutations *xth-* and *nfo-*. *DME* expression in the presence of IPTG is cytotoxic *to E. coli* due to excessive SSBs and 3' blocking ends (red dots) generated by DME. Simultaneous expression of APE and DME maintains the cell growth rate by processing 3' blocking lesions (blue dots). (C) Serial dilution assay to measure cellular toxicity of *DME* expression to AP endonuclease-deficient RPC501 (*xth- nfo-*) strains with increasing amounts of IPTG (0-50 μM). The growth rate of RPC501 decreased as *DME* expression increased due to excessive 5mC excision in the bacterial genome. Unlike *APE1L* and *ARP*, *ZDP* expression did not ameliorate cytotoxicity induced by *DME* expression.

```
                                   * *
xth     : AQFYQNLQNYLETELKRDNPVLIMGDMNISPTDLDIGIGEENRK---------RWLRTGKCSFLPEERE : 185
hAPE1   : QRWDEAFRKFLK-GLASRKPLVLCGDLNVAHEEIDLRNPKGNKK---------N------AGFTPQERQ : 238
AtAPE1L : RKWDKRIVEFLN--KTSDKPLIWCGDLNVSHEEIDVSHPEFFATAKLNGYVPPNKEDCGQPGFTPSERG : 265
AtARP   : IEEWDRTLSNHIKELEKSKPVVLTGDLNCAHEEIDIFNPAGNKR---------S------AGFTIEERQ : 457
hAPE2   : MRFYRLLQIRAEALLAAGSHVIILGDLNTAHRPIDHWDAVNLEC---------FEEDPG--------RK : 223
AtAPE2  : ---------------------------MDRCEAGPDFEKNEFRK------------------------- :  17

                                                                 *
xth     : WMDRLM-SWG---------LVDTFRHAN--PQTADRFSWFDYRSKGFDDNRGLRIDLLLASQPLAECCV : 242
hAPE1   : GFGELLQAVP---------LADSFRHLY--PNTPYAYTFWTYMMNARSKNVGWRLDYFLLSHSLLPALC : 296
AtAPE1L : RFGATIKEGR---------LVDAYRYLHKEQEMESGFSWSGNPIG-KYRGKRMRIDYFLVSEQLKDRIV : 324
AtARP   : SEGANLLDKG---------FVDTFRKQH--P-GVVGYTYWGYRHGGRKTNKGWRLDYFLVSQSIAANVH : 514
hAPE2   : WMDSLLSNLGCQSASHVGPFIDSYRCFQ--PKQEGAFTCWSAVTGARHLNYGSRLDYVLGDRTLVIDTF : 290
AtAPE2  : WFRSLLVERG-------GSFSDVFRSKH--PERKDAFTCWSSSSGAEQFNYGSRIDHILVAGSCLHQDE :  77

                                                       *
xth     : ETGID---Y--------------------------EIRSMEKPSDHAPVWATFRR-------------- : 268
hAPE1   : DSKIR---S--------------------------KA----LGSDHCPITLYLAL-------------- : 318
AtAPE1L : SCKMHG-----------------------------EIRSMEKPSDHAPVWATFRR-------------- : 350
AtARP   : DSYIL---P--------------------------DI----NGSDHCPIGLILKLFVQNFTIPSFLLKS : 550
hAPE2   : QASFL---L--------------------------PE---VMGSDHCPVGAVLSV----SSVPAKQCPP : 323
AtAPE2  : DKQGHSFLACHVKECDILTEYKRFKNENMPTRWKGGLVTKFKGSDHVPVFISFDDL---PDIPEHSTPP : 143
```

Figure S3. Sequence alignment of *E. coli* exonuclease III (Xth) with human hAPE1 and hAPE2, and *Arabidopsis* APE1L, APE2, and ARP.

Conserved residues are colored in black. Amino acid residues required for catalytic activity of AP endonuclease were denoted with asterisks (1).

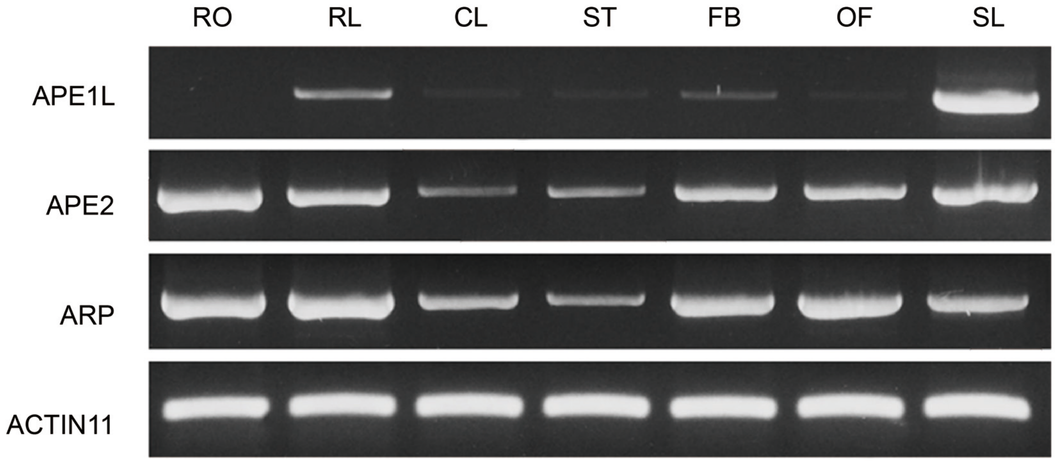

Figure S4. Expression pattern of *APE1L, APE2,* and *ARP* in various tissue types is assessed by quantitative RT-PCR.

ACTIN11 was used as an internal control. RO, root; RL, rosette leaf; CL, cauline leaf; ST, stem; FB, flower bud; OF, open flower; SL, silique.

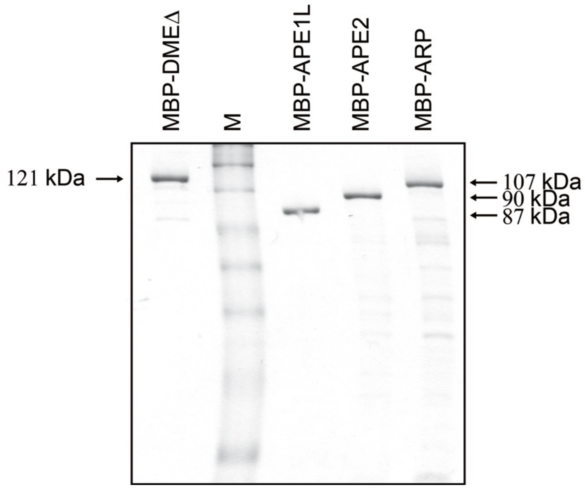

Figure S5. SDS-PAGE gel image of purified *Arabidopsis* AP endonucleases and DME protein fused with an N-terminal MBP.

Two hundred ng of each protein was electrophoresed on a 10% SDS-PAGE gel and visualized by Coomassie Brilliant Blue staining. Expected molecular weights of purified proteins were indicated at each side of the panel. M, size marker.

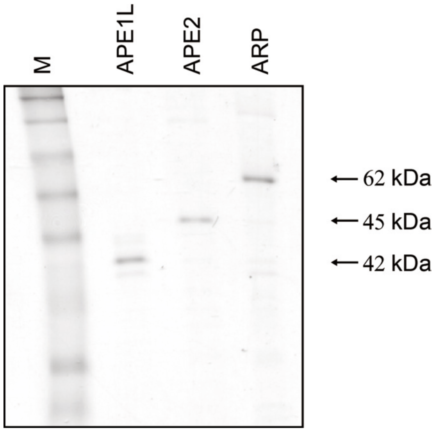

Figure S6. SDS-PAGE gel image of purified *Arabidopsis* AP endonucleases without an MBP. Four hundred ng of each protein was electrophoresed on a 10% SDS-PAGE gel and visualized by Coomassie Brilliant Blue staining. Expected molecular weights of purified proteins were indicated to the right. M, size marker.

The MBP-free AP endonucleases were purified by basically the same experimental procedure as used for MBP-tagged proteins (See supplementary Material and Methods) with minor modifications. Briefly, MBP-tagged APE1L, APE2 and ARP proteins were expressed and purified through the HisTrap FF column. By utilizing the HRV 3C cleavage sequence between MBP and the fusion target, the N-terminal MBP fragment was cleaved off by the treatment with PreScission Protease (GE Healthcare) at 4 °C overnight. The Heparin HP column (GE Healthcare) was used to separate MBP from AP endonuclease fragments. The collected fractions were concentrated and stored at -80 °C until use.

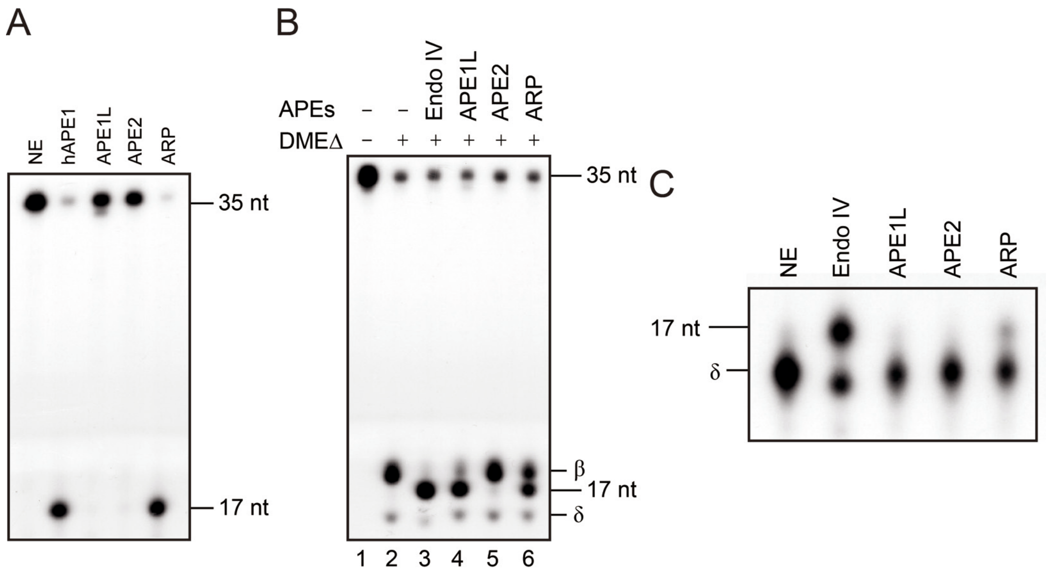

Figure S7. *In vitro* DNA repair activities of *Arabidopsis* MBP-free AP endonucleases.

(A) AP site incision activity of Arabidopsis AP endonucleases. Radiolabeled 35-mer oligonucleotides containing a THF (25 nM) were reacted with 5 nM each of purified APE1L, APE2 and ARP (Figure S6) in the AP endonuclease reaction buffer at 37 °C for 30 min. Sizes of the substrate (35 nt) and the product (17 nt) were indicated to the right. NE, no enzyme; hAPE1, human APE1. (B) The 3'-phosphodiesterase activity on DME-treated products. Radiolabeled 35-mer oligonucleotides containing 5mC (25 nM) were reacted with DME (lane 2), and the reaction product was further incubated with each purified *Arabidopsis* AP endonuclease (Figure S6) in the presence of 2.5 mM $MgCl_2$ (lanes 4-6). The 3' end-processed fragment (17 nt) was indicated relative to DME treated products (β, δ) to the right of the panel. Endo IV, Endonuclease IV. (C) The 3'-phosphatase activity of purified *Arabidopsis* AP endonucleases. The 35-mer oligonucleotide duplex (25 nM) that mimics a DME-catalyzed δ-elimination product was prepared (Figure 5A) and reacted with 5 nM each of APE1L, APE2 and ARP (Figure S6) in the AP endonuclease buffer at 37 °C for 60 min. The DNA substrate (δ) and the product (17 nt) were indicated to the left.

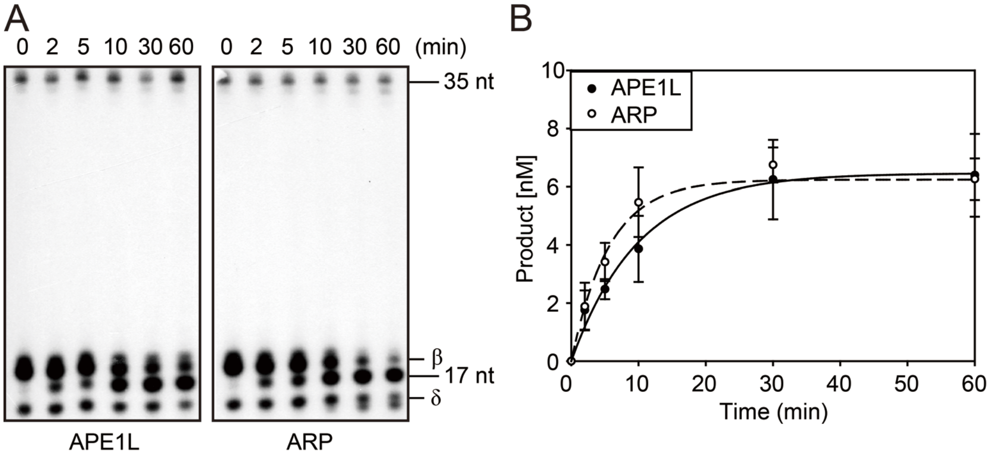

C

Enzyme	P_{max} (%)	T_{50} (min)	E_{rel} (%/min) *
APE1L	43.40 ± 4.42	8.88	4.89 ± 0.50
ARP	41.97 ± 2.93	4.86	8.63 ± 0.60

*E_{rel}, relative processing efficiency (P_{max}/T_{50})

Figure S8. Kinetics analysis for 3'-phosphodiesterase activities of APE1L and ARP.

(A) Radiolabeled DNA duplex containing 5mC (20 nM) was incubated with DME at 37 °C for 1 hr to prepare DNA demethylation products, and then APE1L or ARP (5 nM) was added and reacted for additional time at 37 °C to assess the 3'-phosphodiesterase activities. The reactions were terminated at indicated time points (0, 2, 5, 10, 30, 60 min) and separated on the 15% polyacrylamide gel. The gel was exposed to a phosphorimager screen (Fujifilm) and the radioactivity was measured using the Fujifilm BAS-5000 phosphorimager. The DNA substrate (35 nt) and the end-processed fragment (17 nt) were indicated relative to DME-treated products (β, δ) to the right of the panel. (B) The 3'-end-processed products of APE1L (closed circles) and ARP (open circles) in Figure S8A were quantitated and plotted over time. Error bars represent standard deviations from three independent experiments. (C) In order to compare the 3'-phosphodiesterase activities of APE1L and ARP, the relative processing efficiency (E_{rel}) was determined from the P_{max} (maximum substrate processing within an unlimited period of time)

and T_{50} (the time required to reach 50% of the product plateau level, P_{max}) values. Data from (B) were used to determine the P_{max} and T_{50} values by non-linear regression analysis using the Sigmaplot software. (26,27).

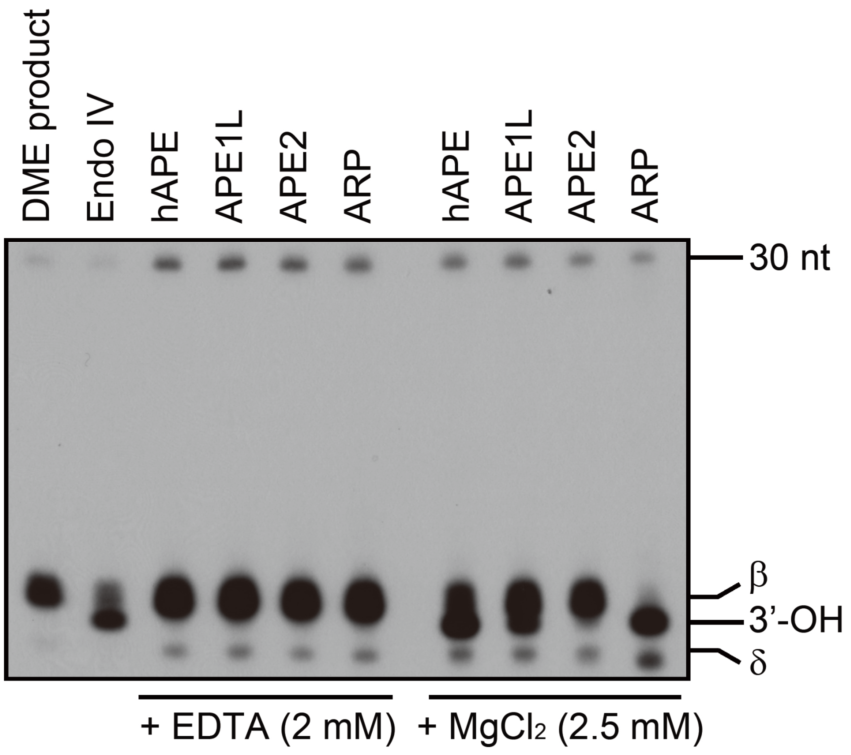

Figure S9. The 3' end processing activity of APE1L, APE2, and ARP on DME 5mC excision products.

Radiolabeled 30-mer DNA duplex with 5mC at position 15 of the top strand (25 nM) was reacted with DME (lane 1), and further incubated with each purified *Arabidopsis* AP endonuclease (5 nM) in the presence of 2 mM EDTA (lanes 3-6) or 2.5 mM $MgCl_2$ (lanes 7-10) at 37 °C for 20 min. Reactions with *E. coli* Endonuclease IV (lane 2) and human hAPE1 (lane 7) were used as controls. The size of AP Endonuclease reaction product (14-nt with 3'-OH) is indicated to the right.

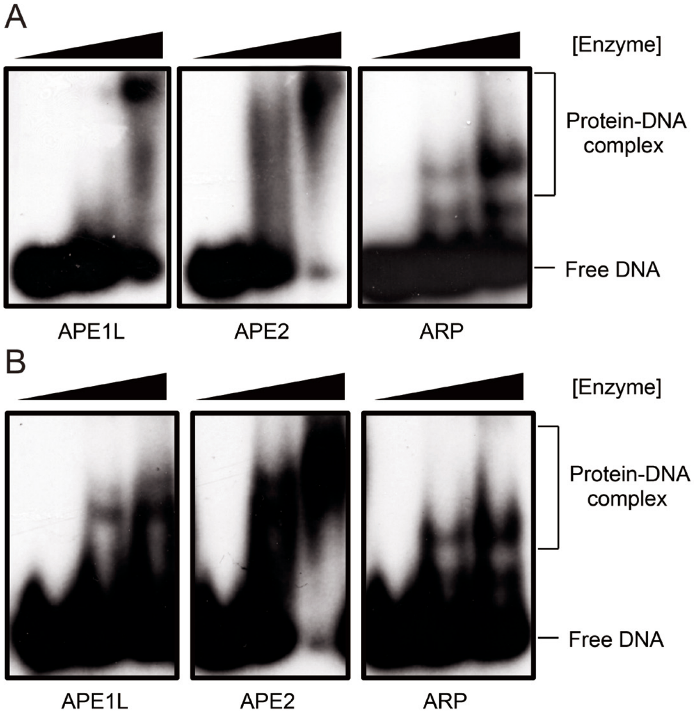

Figure S10. Electrophoretic mobility shift assay of *Arabidopsis* AP endonucleases.

Standard Electrophoretic mobility shift assay was performed as described by Mok et al. (28). Briefly, 50 nM radiolabeled 35-mer oligonucleotides containing a THF (A) or 5mC (B) were incubated with each purified MBP-APE1L, -APE2, or -ARP on ice for 10 min. The increasing amounts of protein (0, 100, and 300 nM) were added to measure DNA binding activity in the DNA binding buffer (10 mM Tris-HCl, pH 8.0, 150 mM NaCl, 0.05% Triton X-100, 0.1 mg/mL BSA, 10% glycerol, and 10 mM DTT). The reactions were separated on the 8% native polyacrylamide gel for 2 hr at 50V and the gel was exposed to an X-ray film. The protein-DNA complex and free DNA were indicated to the right.

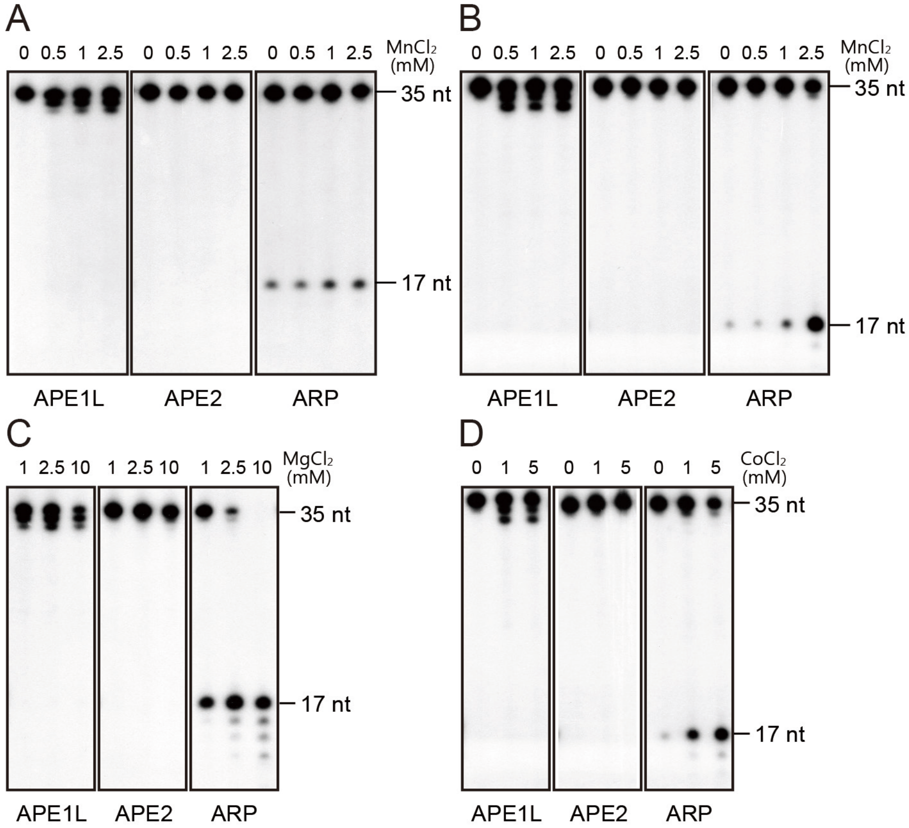

Figure S11. *In vitro* AP site incision activity of *Arabidopsis* AP endonucleases in the presence of various divalent cations.

According to Joldybayeva et al. (29), wheat APE1L (TaAPE1L) was active in the presence of Mn^{2+}, Co^{2+} and Fe^{2+} cations but inhibited by Mg^{2+} and Ca^{2+}. Thus, we tested whether *Arabidopsis* AP endonucleases also had catalytic activity in the reaction condition in which TaAPE1L was active. Briefly, radiolabeled 35-mer oligonucleotide substrates containing a THF (10 nM) were reacted with MBP-APE1L, -APE2, or -ARP in the TaAPE1L reaction buffer (20 mM HEPES-KOH, pH 7.0, 50 mM KCl, 1 mM DTT, 0.1 mg/ml BSA, 0.1% Nonidet P-40) at 23 °C for 5 min. The reactions contained 5 nM (A) or 100 nM (B, C, and D) each of *Arabidopsis* AP endonucleases with various divalent cations: $MnCl_2$ (A and B), $MgCl_2$ (C), and $CoCl_2$ (D). It should be noted that DTT was not included in the presence of $CoCl_2$ to prevent reduction as in the study by Joldybayeva et al. (29) (D). As shown in (A-D), only ARP was able to cleave an AP

site in every condition tested, where APE1L or ARP was still inactive. Notably, unlike TaAPE1L, *Arabidopsis* APE1L did not show discernable AP site incision activity even in the presence of nonionic detergent and $MnCl_2$ or $CoCl_2$ at lower temperature (23 °C), despite significant 3'→5' exonuclease activity observed (A-D). This indicates that *Arabidopsis* APE1L and TaAPE1L have distinct functions and that *Arabidopsis* ARP requires a reaction condition different from that of TaAPE1L for AP site processing. The concentrations of each divalent cation were indicated at the top of the panels and the sizes of the DNA substrate (35 nt) and the product (17 nt) were indicated to the right.

References

1. Mol, C.D., Izumi, T., Mitra, S. and Tainer, J.A. (2000) DNA-bound structures and mutants reveal abasic DNA binding by APE1 and DNA repair coordination. *Nature*, **403**, 451-456.
2. Chou, K.M. and Cheng, Y.C. (2003) The exonuclease activity of human apurinic/apyrimidinic endonuclease (APE1). *J. Biol. Chem.*, **278**, 18289-18296.
3. Gros, L., Ishchenko, A.A., Ide, H., Elder, R.H. and Saparbaev, M.K. (2004) The major human AP endonuclease (Ape1) is involved in the nucleotide incision repair pathway. *Nucleic Acids Res.*, **32**, 73-81.
4. Castillo-Acosta, V.M., Ruiz-Pérez, L.M., Yang, W., González-Pacanowska, D. and Vidal, A.E. (2009) Identification of a residue critical for the excision of 3'-blocking ends in apurinic/apyrimidinic endonucleases of the Xth family. *Nucleic Acids Res.*, **37**, 1829-1842.
5. Lu, D., Silhan, J., MacDonald, J.T., Carpenter, E.P., Jensen, K., Tang, C.M., Baldwin, G.S. and Freemont, P.S. (2012) Structural basis for the recognition and cleavage of abasic DNA in *Neisseria meningitidis*. *Proc. Natl. Acad. Sci. USA*, **109**, 16852-16857.
6. Córdoba-Cañero, D., Dubois, E., Ariza, R.R., Doutriaux, M.P. and Roldán-Arjona, T. (2010) *Arabidopsis* uracil DNA glycosylase (UNG) is required for base excision repair of uracil and increases plant sensitivity to 5-fluorouracil. *J. Biol. Chem.*, **285**, 7475-7483.
7. Gao, M.J. and Murphy, T.M. (2001) Alternative forms of formamidopyrimidine-DNA glycosylase from *Arabidopsis thaliana*. *Photochem. Photobiol.*, **73**, 128-134.
8. Murphy, T.M. and Gao, M.J. (2001) Multiple forms of formamidopyrimidine-DNA glycosylase produced by alternative splicing in *Arabidopsis thaliana*. *J. Photochem. Photobiol. B*, **61**, 87-93.

9. Murphy, T.M. and George, A. (2005) A comparison of two DNA base excision repair glycosylases from *Arabidopsis thaliana*. *Biochem. Biophys. Res. Commun.*, **329**, 869-872.

10. Kathe, S.D., Barrantes-Reynolds, R., Jaruga, P., Newton, M.R., Burrows, C.J., Bandaru, V., Dizdaroglu, M., Bond, J.P. and Wallace, S.S. (2009) Plant and fungal Fpg homologs are formamidopyrimidine DNA glycosylases but not 8-oxoguanine DNA glycosylases. *DNA Repair*, **8**, 643-653.

11. Ohtsubo, T., Matsuda, O., Iba, K., Terashima, I., Sekiguchi, M. and Nakabeppu, Y. (1998) Molecular cloning of *AtMMH*, an *Arabidopsis thaliana* ortholog of the *Escherichia coli mutM* gene, and analysis of functional domains of its product. *Mol. Gen. Genet.*, **259**, 577-590.

12. Chen, H., Chu, P., Zhou, Y., Li, Y., Liu, J., Ding, Y., Tsang, E.W., Jiang, L., Wu, K. and Huang, S. (2012) Overexpression of *AtOGG1*, a DNA glycosylase/AP lyase, enhances seed longevity and abiotic stress tolerance in *Arabidopsis*. *J. Exp. Bot.*, **63**, 4107-4121.

13. Morales-Ruiz, T., Birincioglu, M., Jaruga, P., Rodriguez, H., Roldán-Arjona, T. and Dizdaroglu, M. (2003) *Arabidopsis thaliana* Ogg1 protein excises 8-hydroxyguanine and 2,6-diamino-4-hydroxy-5-formamidopyrimidine from oxidatively damaged DNA containing multiple lesions. *Biochemistry*, **42**, 3089-3095.

14. Dany, A.L. and Tissier, A. (2001) A functional OGG1 homologue from *Arabidopsis thaliana. Mol. Genet. Genomics*, **265**, 293-301.

15. García-Ortiz, M.V., Ariza, R.R. and Roldán-Arjona, T. (2001) An *OGG1* orthologue encoding a functional 8-oxoguanine DNA glycosylase/lyase in *Arabidopsis thaliana*. *Plant Mol. Biol.*, **47**, 795-804.

16. Gutman, B.L. and Niyogi, K.K. (2009) Evidence for base excision repair of oxidative DNA damage in chloroplasts of *Arabidopsis thaliana*. *J. Biol. Chem.*, **284**, 17006-17012.

17. Choi, Y., Gehring, M., Johnson, L., Hannon, M., Harada, J.J., Goldberg, R.B., Jacobsen, S.E. and Fischer, R.L. (2002) DEMETER, a DNA glycosylase domain protein, is required for endosperm gene imprinting and seed viability in *Arabidopsis*. *Cell*, **110**, 33-42.

18. Gehring, M., Huh, J.H., Hsieh, T.F., Penterman, J., Choi, Y., Harada, J.J., Goldberg, R.B. and Fischer, R.L. (2006) DEMETER DNA glycosylase establishes *MEDEA* polycomb gene self-imprinting by allele-specific demethylation. *Cell*, **124**, 495-506.

19. Penterman, J., Zilberman, D., Huh, J.H., Ballinger, T., Henikoff, S. and Fischer, R.L. (2007) DNA demethylation in the *Arabidopsis* genome. *Proc. Natl. Acad. Sci. USA,* **104**, 6752-6757.

20. Jang, H., Shin, H., Eichman, B.F. and Huh, J.H. (2014) Excision of 5-hydroxymethylcytosine by DEMETER family DNA glycosylases. *Biochem. Biophys. Res. Commun.*, **446**, 1067-1072.

21. Brooks, S.C., Fischer, R.L., Huh, J.H. and Eichman, B.F. (2014) 5-Methylcytosine Recognition by *Arabidopsis thaliana* DNA Glycosylases DEMETER and DML3. *Biochemistry*, **53**, 2525-2532.

22. Gong, Z., Morales-Ruiz, T., Ariza, R.R., Roldán-Arjona, T., David, L. and Zhu, J.K. (2002) *ROS1*, a repressor of transcriptional gene silencing in *Arabidopsis*, encodes a DNA glycosylase/lyase. *Cell*, **111**, 803-814.

23. Agius, F., Kapoor, A. and Zhu, J.K. (2006) Role of the *Arabidopsis* DNA glycosylase/lyase ROS1 in active DNA demethylation. *Proc. Natl. Acad. Sci. USA*, **103**, 11796-11801.

24. Ponferrada-Marín, M.I., Roldán-Arjona, T. and Ariza, R.R. (2009) ROS1 5-methylcytosine DNA glycosylase is a slow-turnover catalyst that initiates DNA demethylation in a distributive fashion. *Nucleic Acids Res.*, **37**, 4264-4274.

25. Ramiro-Merina, A., Ariza, R.R. and Roldán-Arjona, T. (2013) Molecular characterization of a putative plant homolog of MBD4 DNA glycosylase. *DNA Repair*, **12**, 890-898.

26. Hardeland, U., Bentele, M., Jiricny, J. and Schar, P. (2000) Separating substrate recognition from base hydrolysis in human thymine DNA glycosylase by mutational analysis. *J. Biol. Chem.*, **275**, 33449-33456.

27. Ponferrada-Marín, M.I., Roldán-Arjona, T. and Ariza, R.R. (2012) Demethylation initiated by ROS1 glycosylase involves random sliding along DNA. *Nucleic Acids Res.*, **40**, 11554-11562.

28. Mok, Y.G., Uzawa, R., Lee, J., Weiner, G.M., Eichman, B.F., Fischer, R.L. and Huh, J.H. (2010) Domain structure of the DEMETER 5-methylcytosine DNA glycosylase. *Proc. Natl. Acad. Sci. USA*, **107**, 19225-19230.

29. Joldybayeva, B., Prorok, P., Grin, I.R., Zharkov, D.O., Ishenko, A.A., Tudek, B., Bissenbaev, A.K. and Saparbaev, M. (2014) Cloning and characterization of a wheat homologue of apurinic/apyrimidinic endonuclease Ape1L. *PloS One*, **9**, e92963.
